# Supplementary material for: Autoimmune Disease Classification Based on PubMed Text Mining
Source: J Clin Med. 2022 Jul 26;11(15):4345. doi: 10.3390/jcm11154345 (PMC9369164; doi:10.3390/jcm11154345)
Supplement: Supplementary file 1 [file jcm-11-04345-s001.zip › jcm-1744611-supplementary Table S1.pdf]

	Addison's disease	Still's disease	Alopecia areata	Amyloidosis
Addison's disease	1.000000000	0.000000000	0.001863065	0.000356086
Still's disease	0.000000000	1.000000000	0.000000000	0.000303435
Alopecia areata	0.001863065	0.000000000	1.000000000	0.000187156
Amyloidosis	0.000356086	0.000303435	0.000187156	1.000000000
Ankylosing spondylitis	0.000339789	0.000300135	0.000532220	0.003892319
Antiphospholipid syndrome	0.001165901	0.000181110	0.000258381	0.000391831
Autoimmune angioedema	0.000000000	0.000000000	0.000000000	0.000000000
Autoimmune dysautonomia	0.000000000	0.000000000	0.000000000	0.000000000
Autoimmune encephalomyelitis	0.000000000	0.000000000	0.000107712	0.000043000
Autoimmune hepatitis	0.002503129	0.000729076	0.000531256	0.000357106
Autoimmune inner ear disease	0.000000000	0.000000000	0.000000000	0.000000000
Autoimmune myocarditis	0.000000000	0.000000000	0.000000000	0.000000000
Autoimmune oophoritis	0.002024804	0.000000000	0.000000000	0.000000000
Autoimmune orchitis	0.000000000	0.000000000	0.000000000	0.000000000
Autoimmune pancreatitis	0.000000000	0.000000000	0.000000000	0.000114548
Autoimmune retinopathy	0.000000000	0.000000000	0.000000000	0.000000000
Bullous pemphigoid	0.000000000	0.000000000	0.002643172	0.000262116
Castleman disease	0.000000000	0.000592417	0.000000000	0.001822055
Celiac disease	0.003115829	0.000000000	0.001296562	0.001276121
Cicatricial pemphigoid	0.000000000	0.000000000	0.000000000	0.000000000
Cogan's syndrome	0.000000000	0.000000000	0.000000000	0.000000000
Cold agglutinin disease	0.000000000	0.000000000	0.000000000	0.000362439
CREST syndrome	0.000000000	0.000000000	0.000000000	0.000149638
Crohn's disease	0.000332426	0.000044900	0.000571359	0.002422821
Dermatitis herpetiformis	0.000561877	0.000000000	0.002754131	0.000250536
Discoid lupus	0.000401553	0.000000000	0.008078129	0.000330843
Dressler's syndrome	0.000000000	0.000000000	0.000000000	0.000000000
Endometriosis	0.000120861	0.000000000	0.000000000	0.000306996
Eosinophilic esophagitis	0.000000000	0.000000000	0.000000000	0.000000000
Eosinophilic fasciitis	0.000000000	0.000000000	0.000000000	0.000089800
Erythema nodosum	0.000235766	0.000405762	0.001282051	0.000751355
Essential mixed cryoglobulinemia	0.000000000	0.000000000	0.000000000	0.000000000
Evans syndrome	0.000000000	0.000000000	0.000000000	0.000060100
Fibromyalgia	0.000311003	0.000000000	0.000000000	0.000178241
Fibrosing alveolitis	0.000000000	0.000000000	0.000000000	0.000059600
Glomerulonephritis	0.000197765	0.000070000	0.000000000	0.011048226
Goodpasture's syndrome	0.000423549	0.000000000	0.000000000	0.000149129
Granulomatosis with Polyangiitis	0.000242994	0.000000000	0.000000000	0.000680768
Graves disease	0.005709096	0.000000000	0.001119728	0.000134473
Guillain-Barre syndrome	0.000491125	0.000000000	0.000132057	0.000627294
Hashimoto's thyroiditis	0.009828904	0.000000000	0.002940536	0.000215500
Hemolytic anemia	0.000169342	0.000077000	0.000131518	0.000411551
Henoch-Schonlein purpura	0.000300436	0.000000000	0.000795756	0.000592434
Interstitial cystitis	0.000000000	0.000000000	0.000000000	0.000109923
Kawasaki disease	0.000000000	0.000000000	0.000349864	0.000254136
Lambert-Eaton syndrome	0.000000000	0.000000000	0.000000000	0.000000000

Leukocytoclastic vasculitis	0.000288060	0.000000000	0.000766675	0.000363728
Lichen planus	0.000354887	0.000000000	0.011409668	0.002078662
Lichen sclerosus	0.000000000	0.000000000	0.002694328	0.000141395
Ligneous conjunctivitis	0.000000000	0.000000000	0.000000000	0.000121766
Lupus	0.000833779	0.000149477	0.002001634	0.003511174
Meniere's disease	0.000000000	0.000000000	0.000000000	0.000000000
Microscopic polyangiitis	0.000348797	0.000000000	0.000000000	0.000173651
Mixed connective tissue disease	0.000000000	0.001068376	0.000275596	0.000284269
Mucha-Habermann disease	0.000000000	0.000000000	0.000000000	0.000000000
Multifocal Motor Neuropathy	0.000000000	0.000000000	0.000000000	0.000089600
Multiple sclerosis	0.000326023	0.000000000	0.000301357	0.000422116
Myasthenia gravis	0.001885691	0.000000000	0.001251132	0.000391252
Narcolepsy	0.000308356	0.000000000	0.000376612	0.000051900
Neonatal Lupus	0.000000000	0.000000000	0.000000000	0.000000000
Neuromyelitis optica	0.000000000	0.000000000	0.000000000	0.000000000
Neutropenia	0.000055400	0.000058300	0.000109472	0.000743008
Paroxysmal nocturnal hemoglobinuria	0.000000000	0.000000000	0.000000000	0.000056900
Parry Romberg syndrome	0.000000000	0.000000000	0.000000000	0.000000000
Parsonage-Turner syndrome	0.000000000	0.000000000	0.000000000	0.000000000
Pemphigus	0.000408024	0.000000000	0.003408141	0.000436631
Peripheral neuropathy	0.000510421	0.000100170	0.000081900	0.007235082
Pernicious anemia	0.008751229	0.000000000	0.001886623	0.000102359
POEMS syndrome	0.000781861	0.000000000	0.000000000	0.002273667
Polyarteritis nodosa	0.000733003	0.000543257	0.000253979	0.002092683
Polyglandular syndromes type I	0.034435262	0.000000000	0.002041574	0.000000000
Polyglandular syndromes type II	0.013615734	0.000000000	0.000000000	0.000000000
Polyglandular syndromes type III	0.003067485	0.000000000	0.000415369	0.000000000
Polymyalgia rheumatica	0.000692137	0.001908917	0.000369504	0.000943972
Polymyositis	0.000727846	0.000463750	0.000632875	0.001436242
Primary biliary cirrhosis	0.001298252	0.000000000	0.000240790	0.000594530
Primary sclerosing cholangitis	0.000224770	0.000000000	0.000408622	0.000583539
Progesterone dermatitis	0.000000000	0.000000000	0.000000000	0.000000000
Psoriasis	0.000293407	0.000036800	0.007895323	0.001477173
Psoriatic arthritis	0.000204082	0.000538503	0.002120414	0.001196117
Pure red cell aplasia	0.000000000	0.002370792	0.000457596	0.000115992
Pyoderma gangrenosum	0.000000000	0.000513875	0.002282556	0.000524080
Raynaud's phenomenon	0.000234494	0.000402091	0.000000000	0.000267773
Relapsing polychondritis	0.000000000	0.000000000	0.000465622	0.000844324
Retroperitoneal fibrosis	0.000000000	0.000000000	0.000000000	0.000503229
Rheumatic fever	0.000299706	0.000152311	0.000000000	0.000483623
Rheumatoid arthritis	0.000562845	0.000339074	0.000699257	0.006869878
Sarcoidosis	0.000533079	0.000218075	0.000602195	0.005206466
Scleritis	0.000000000	0.000815993	0.000000000	0.000143567
Scleroderma	0.000474774	0.000071300	0.001695020	0.002438377
Sjogren's syndrome	0.001348943	0.000348351	0.001015838	0.004535790
Stiff person syndrome	0.000417362	0.000000000	0.000000000	0.000000000
Susac's syndrome	0.000000000	0.000000000	0.000000000	0.000000000

Sympathetic ophthalmia	0.000000000	0.000000000	0.000000000	0.000000000
Takayasu's arteritis	0.000000000	0.000941176	0.000000000	0.000521890
Thrombocytopenic purpura	0.000252823	0.000247807	0.000406157	0.000494889
Thyroid eye disease	0.000000000	0.000000000	0.000000000	0.000000000
Transverse myelitis	0.000000000	0.000000000	0.000000000	0.000000000
Ulcerative colitis	0.000293277	0.000039200	0.000559224	0.001585300
Undifferentiated connective tissue disease	0.000000000	0.000000000	0.000000000	0.000060500
Uveitis	0.000070800	0.000205968	0.000623974	0.002068005
Vasculitis	0.000207250	0.000187084	0.000282810	0.004981450
Vitiligo	0.008277027	0.000000000	0.033258174	0.000269925
Vogt-Koyanagi-Harada Disease	0.000000000	0.000000000	0.000000000	0.000000000

Ankylosing spondylitis	Antiphospholipid syndrome	Autoimmune angioedema	Autoimmune dysautonomia
0.000339789	0.001165901	0.000000000	0.000000000
0.000300135	0.000181110	0.000000000	0.000000000
0.000532198	0.000258365	0.000000000	0.000000000
0.003892319	0.000391831	0.000000000	0.000000000
1.000000000	0.000887953	0.000000000	0.000000000
0.000887953	1.000000000	0.000000000	0.000000000
0.000000000	0.000000000	1.000000000	0.000000000
0.000000000	0.000000000	0.000000000	1.000000000
0.000059700	0.000000000	0.000000000	0.000000000
0.000457369	0.003132069	0.000000000	0.000000000
0.000000000	0.000182999	0.000000000	0.000000000
0.000000000	0.000000000	0.000000000	0.000000000
0.000000000	0.000000000	0.000000000	0.000000000
0.000000000	0.000000000	0.000000000	0.000000000
0.000000000	0.000307763	0.000000000	0.000000000
0.000000000	0.000000000	0.000000000	0.000000000
0.000119100	0.000246396	0.000000000	0.000000000
0.000087800	0.000144781	0.000000000	0.000000000
0.001426534	0.000752663	0.000000000	0.000000000
0.000096900	0.000000000	0.000000000	0.000000000
0.000199193	0.000269590	0.000000000	0.000000000
0.000000000	0.000178635	0.000000000	0.000000000
0.000146908	0.000349040	0.000000000	0.000000000
0.009084027	0.000436308	0.000000000	0.000000000
0.000392140	0.000000000	0.000000000	0.000000000
0.000729802	0.001394895	0.000000000	0.000000000
0.000000000	0.000000000	0.000000000	0.000000000
0.000143062	0.000200120	0.000000000	0.000000000
0.000000000	0.000000000	0.000000000	0.000000000
0.000195618	0.000173958	0.000000000	0.000000000
0.002886360	0.000455670	0.000000000	0.000000000
0.000000000	0.000181686	0.000000000	0.000000000
0.000000000	0.001676668	0.000000000	0.000000000
0.005766322	0.000479198	0.000000000	0.000000000
0.000194354	0.000430034	0.000000000	0.000000000
0.001190538	0.003653755	0.000000000	0.000000000
0.000194581	0.000344709	0.000000000	0.000000000
0.001101048	0.001666580	0.000000000	0.000000000
0.000614345	0.000730533	0.000000000	0.000000000
0.000232635	0.000804658	0.000000000	0.000000000
0.000331249	0.000855657	0.000000000	0.000000000
0.000529287	0.004967573	0.000000000	0.000000000
0.000355745	0.001034814	0.000000000	0.000000000
0.000000000	0.000000000	0.000000000	0.000000000
0.000303272	0.000918590	0.000000000	0.000000000
0.000000000	0.000000000	0.000000000	0.000000000

0.000570952	0.001158329	0.000000000	0.000000000
0.000233910	0.000286068	0.000000000	0.000000000
0.000000000	0.000000000	0.000000000	0.000000000
0.000000000	0.000000000	0.000000000	0.000000000
0.008599158	0.054019579	0.000000000	0.000000000
0.000117679	0.000120948	0.000000000	0.000000000
0.000092700	0.001030764	0.000000000	0.000000000
0.002391697	0.004699462	0.000000000	0.000000000
0.000000000	0.000000000	0.000000000	0.000000000
0.000097500	0.000000000	0.000000000	0.000000000
0.001726875	0.001335965	0.000000000	0.000000000
0.000944238	0.001131260	0.000000000	0.000000000
0.000156403	0.000000000	0.000000000	0.000000000
0.000000000	0.003560264	0.000000000	0.000000000
0.000244698	0.001350743	0.000000000	0.000000000
0.000409072	0.000594673	0.000000000	0.000000000
0.000090200	0.001516300	0.000000000	0.000000000
0.000000000	0.000000000	0.000000000	0.000000000
0.000000000	0.000000000	0.000000000	0.000000000
0.000392850	0.000370508	0.000000000	0.000000000
0.000304901	0.000559321	0.000000000	0.000000000
0.000536563	0.000174978	0.000000000	0.000000000
0.000000000	0.000000000	0.000000000	0.000000000
0.002734492	0.002645800	0.000000000	0.000000000
0.000000000	0.000000000	0.000000000	0.000000000
0.000000000	0.000000000	0.000000000	0.000000000
0.000000000	0.000000000	0.000000000	0.000000000
0.004877199	0.000708818	0.000000000	0.000000000
0.004502966	0.002700965	0.000000000	0.000000000
0.000547425	0.001682314	0.000000000	0.000000000
0.001254248	0.000506939	0.000000000	0.000000000
0.000000000	0.000000000	0.000000000	0.000000000
0.029962181	0.000354812	0.000000000	0.000000000
0.075855754	0.001019935	0.000000000	0.000000000
0.000093000	0.000318700	0.000000000	0.000000000
0.002625350	0.001396746	0.000000000	0.000000000
0.000492490	0.004494528	0.000000000	0.000000000
0.001403903	0.001045941	0.000000000	0.000000000
0.000877578	0.000216606	0.000000000	0.000000000
0.006247487	0.000933945	0.000000000	0.000000000
0.032497861	0.002110343	0.000000000	0.000000000
0.003898028	0.000870302	0.000000000	0.000000000
0.001649938	0.000543015	0.000000000	0.000000000
0.004891477	0.004435284	0.000000000	0.000000000
0.006097226	0.006802969	0.000000000	0.000000000
0.000000000	0.000171204	0.000000000	0.000000000
0.000000000	0.000183672	0.000000000	0.000000000

0.000386847	0.000000000	0.000000000	0.000000000
0.000697156	0.000955338	0.000000000	0.000000000
0.000404347	0.007201815	0.000000000	0.000000000
0.000000000	0.000000000	0.000000000	0.000000000
0.000222797	0.004692388	0.000000000	0.000000000
0.007050210	0.000358073	0.000000000	0.000000000
0.000299043	0.001983769	0.000000000	0.000000000
0.022072112	0.001157642	0.000000000	0.000000000
0.003934186	0.006050271	0.000000000	0.000000000
0.000828441	0.000371925	0.000000000	0.000000000
0.000244081	0.000000000	0.000000000	0.000000000

Autoimmune encephalomyelitis	Autoimmune hepatitis	Autoimmune inner ear disease	Autoimmune myocarditis
0.000000000	0.002503129	0.000000000	0.000000000
0.000000000	0.000729076	0.000000000	0.000000000
0.000107706	0.000531208	0.000000000	0.000000000
0.000043000	0.000357106	0.000000000	0.000000000
0.000059700	0.000457369	0.000000000	0.000000000
0.000000000	0.003132069	0.000182999	0.000000000
0.000000000	0.000000000	0.000000000	0.000000000
0.000000000	0.000000000	0.000000000	0.000000000
1.000000000	0.000245399	0.000000000	0.000549187
0.000245399	1.000000000	0.000000000	0.000410846
0.000000000	0.000000000	1.000000000	0.000000000
0.000549187	0.000410846	0.000000000	1.000000000
0.000000000	0.000000000	0.000000000	0.000000000
0.000285144	0.000000000	0.000000000	0.000000000
0.000000000	0.004787416	0.000000000	0.000000000
0.000000000	0.000000000	0.000000000	0.000000000
0.000000000	0.000165975	0.000000000	0.000000000
0.000000000	0.000000000	0.000000000	0.000000000
0.000159367	0.004861593	0.000000000	0.000244170
0.000000000	0.000000000	0.000000000	0.000000000
0.000000000	0.000000000	0.017513135	0.000000000
0.000000000	0.000000000	0.000000000	0.000000000
0.000000000	0.001789401	0.000000000	0.000000000
0.000395910	0.001891663	0.000089900	0.000000000
0.000000000	0.000611309	0.000000000	0.000000000
0.000000000	0.000295014	0.000000000	0.000000000
0.000000000	0.000000000	0.000000000	0.000000000
0.000046400	0.000000000	0.000000000	0.000000000
0.000000000	0.000000000	0.000000000	0.000000000
0.000000000	0.000000000	0.000000000	0.000000000
0.000000000	0.000536817	0.000000000	0.000000000
0.000000000	0.000000000	0.000000000	0.000000000
0.000000000	0.000977927	0.000000000	0.000000000
0.000076800	0.000159770	0.000000000	0.000000000
0.000000000	0.000268673	0.000000000	0.000000000
0.000226488	0.000821018	0.000052600	0.000052100
0.000000000	0.000000000	0.000000000	0.000000000
0.000000000	0.000465426	0.000000000	0.000000000
0.000000000	0.001582401	0.000000000	0.000248262
0.001115564	0.000412955	0.000000000	0.000000000
0.000000000	0.002456108	0.000429830	0.000575705
0.000177242	0.001958590	0.000000000	0.000000000
0.000000000	0.000213789	0.000000000	0.000000000
0.000000000	0.000000000	0.000000000	0.000000000
0.000000000	0.000377558	0.000000000	0.000000000
0.000000000	0.000000000	0.000000000	0.000000000

0.000000000	0.000622600	0.000000000	0.000000000
0.000000000	0.001311866	0.000000000	0.000000000
0.000000000	0.000000000	0.000000000	0.000000000
0.000000000	0.000000000	0.000000000	0.000000000
0.001494782	0.003635816	0.000074800	0.000106258
0.000000000	0.000000000	0.003859708	0.000000000
0.000000000	0.000711406	0.000000000	0.000000000
0.000000000	0.002653106	0.000000000	0.000000000
0.000000000	0.000000000	0.000000000	0.000000000
0.000000000	0.000000000	0.000000000	0.000000000
0.079644426	0.000866167	0.000033900	0.000112448
0.001519898	0.001240595	0.000000000	0.000312484
0.000101507	0.000000000	0.000000000	0.000000000
0.000000000	0.000271370	0.000000000	0.000000000
0.004740358	0.000351525	0.000000000	0.000000000
0.000328711	0.000561085	0.000000000	0.000029000
0.000000000	0.000000000	0.000000000	0.000000000
0.000000000	0.000000000	0.000000000	0.000000000
0.000000000	0.000000000	0.000000000	0.000000000
0.000000000	0.000747041	0.000000000	0.000000000
0.000298641	0.000228833	0.000000000	0.000000000
0.000000000	0.001158480	0.000000000	0.000000000
0.000000000	0.000255689	0.000000000	0.000000000
0.000095700	0.001102050	0.000000000	0.000000000
0.000000000	0.005018121	0.000000000	0.000000000
0.000000000	0.000595238	0.000000000	0.000000000
0.000000000	0.000301841	0.000000000	0.000000000
0.000116279	0.000402860	0.000000000	0.000000000
0.000151126	0.001825960	0.000000000	0.000149376
0.000139282	0.074531356	0.000000000	0.000236435
0.000000000	0.063318977	0.000000000	0.000000000
0.000000000	0.000000000	0.000000000	0.000000000
0.000766080	0.000676344	0.000036800	0.000054700
0.000121590	0.000920016	0.000181258	0.000000000
0.000000000	0.000478183	0.000000000	0.000000000
0.000000000	0.001479582	0.000000000	0.000000000
0.000000000	0.001515422	0.000000000	0.000000000
0.000000000	0.000000000	0.002126528	0.000000000
0.000000000	0.000310430	0.000000000	0.000000000
0.000075000	0.000000000	0.000000000	0.000220962
0.001605855	0.001222866	0.000014400	0.000050300
0.000087700	0.000860900	0.000000000	0.000122892
0.000000000	0.000229621	0.000000000	0.000000000
0.000192428	0.001254742	0.000000000	0.000000000
0.000227635	0.005694468	0.000116850	0.000113154
0.000203197	0.000000000	0.000000000	0.000000000
0.000000000	0.000000000	0.000000000	0.000000000

0.000000000	0.000000000	0.000000000	0.000000000
0.000000000	0.000000000	0.000000000	0.000000000
0.000059300	0.001666793	0.000000000	0.000000000
0.000000000	0.000000000	0.000000000	0.000000000
0.000543380	0.000215031	0.000000000	0.000000000
0.000263452	0.002695984	0.000078600	0.000000000
0.000000000	0.000000000	0.000000000	0.000000000
0.001912851	0.000598909	0.000051600	0.000050900
0.000174913	0.000810578	0.000107003	0.000017700
0.000091300	0.002327492	0.000000000	0.000000000
0.000000000	0.000000000	0.000000000	0.000000000

Autoimmune oophoritis	Autoimmune orchitis	Autoimmune pancreatitis	Autoimmune retinopathy
0.002024804	0.000000000	0.000000000	0.000000000
0.000000000	0.000000000	0.000000000	0.000000000
0.000000000	0.000000000	0.000000000	0.000000000
0.000000000	0.000000000	0.000114557	0.000000000
0.000000000	0.000000000	0.000000000	0.000000000
0.000000000	0.000000000	0.000307811	0.000000000
0.000000000	0.000000000	0.000000000	0.000000000
0.000000000	0.000000000	0.000000000	0.000000000
0.000000000	0.000285164	0.000000000	0.000000000
0.000000000	0.000000000	0.004787962	0.000000000
0.000000000	0.000000000	0.000000000	0.000000000
0.000000000	0.000000000	0.000000000	0.000000000
1.000000000	0.006557377	0.000000000	0.000000000
0.006557377	1.000000000	0.000000000	0.000000000
0.000000000	0.000000000	1.000000000	0.000000000
0.000000000	0.000000000	0.000000000	1.000000000
0.000000000	0.000000000	0.000000000	0.000000000
0.000000000	0.000000000	0.002066116	0.000000000
0.000000000	0.000000000	0.000383450	0.000000000
0.000000000	0.000000000	0.000000000	0.000000000
0.000000000	0.000000000	0.000000000	0.000000000
0.000000000	0.000000000	0.000000000	0.000000000
0.000000000	0.000000000	0.000000000	0.000000000
0.000000000	0.000000000	0.000666409	0.000000000
0.000000000	0.000000000	0.000000000	0.000000000
0.000000000	0.000000000	0.000000000	0.000000000
0.000000000	0.000000000	0.000000000	0.000000000
0.000000000	0.000000000	0.000000000	0.000000000
0.000000000	0.000000000	0.000379219	0.000000000
0.000000000	0.000000000	0.000000000	0.000000000
0.000000000	0.000000000	0.000000000	0.000000000
0.000000000	0.000000000	0.000000000	0.000000000
0.000000000	0.000000000	0.000000000	0.000000000
0.000000000	0.000000000	0.000000000	0.000000000
0.000000000	0.000000000	0.000000000	0.000000000
0.000000000	0.000035100	0.000253906	0.000000000
0.000000000	0.000000000	0.000000000	0.000000000
0.000000000	0.000000000	0.000279122	0.000000000
0.000000000	0.000000000	0.000138555	0.000000000
0.000000000	0.000000000	0.000000000	0.000000000
0.000000000	0.000000000	0.000446362	0.000000000
0.000000000	0.000000000	0.000214846	0.000000000
0.000000000	0.000000000	0.000000000	0.000000000
0.000000000	0.000000000	0.000000000	0.000000000
0.000000000	0.000000000	0.000000000	0.000000000
0.000000000	0.000000000	0.000000000	0.000000000

0.000000000	0.000000000	0.000561377	0.000000000
0.000000000	0.000000000	0.000000000	0.000000000
0.000000000	0.000000000	0.000000000	0.000000000
0.000000000	0.000000000	0.000000000	0.000000000
0.000053500	0.000021400	0.000209181	0.000042800
0.000000000	0.000000000	0.000000000	0.000000000
0.000000000	0.000000000	0.000000000	0.000000000
0.000000000	0.000000000	0.000000000	0.000000000
0.000000000	0.000000000	0.000000000	0.000000000
0.000000000	0.000000000	0.000000000	0.000000000
0.000000000	0.000000000	0.000033200	0.000034000
0.000000000	0.000000000	0.000241371	0.000000000
0.000000000	0.000000000	0.000000000	0.000000000
0.000000000	0.000000000	0.000000000	0.000000000
0.000000000	0.000000000	0.000000000	0.000000000
0.000000000	0.000000000	0.000000000	0.000000000
0.000000000	0.000000000	0.000000000	0.000000000
0.000000000	0.000000000	0.000000000	0.000000000
0.000000000	0.000000000	0.000000000	0.000000000
0.000000000	0.000000000	0.000305064	0.000000000
0.000000000	0.000000000	0.000000000	0.000000000
0.000000000	0.000000000	0.000000000	0.000000000
0.000000000	0.000000000	0.000000000	0.000000000
0.000000000	0.000000000	0.000000000	0.000000000
0.000000000	0.000000000	0.000000000	0.000000000
0.000000000	0.000000000	0.000000000	0.000000000
0.000000000	0.000000000	0.000000000	0.000000000
0.000000000	0.000000000	0.000000000	0.000000000
0.000000000	0.000000000	0.000000000	0.000000000
0.000000000	0.000000000	0.002512815	0.000000000
0.000000000	0.000000000	0.020536463	0.000000000
0.000000000	0.000000000	0.000000000	0.000000000
0.000000000	0.000000000	0.000053200	0.000000000
0.000000000	0.000000000	0.000000000	0.000000000
0.000000000	0.000000000	0.000000000	0.000000000
0.000000000	0.000000000	0.000000000	0.000000000
0.000000000	0.000000000	0.000000000	0.000000000
0.000000000	0.000000000	0.000000000	0.000000000
0.000000000	0.000000000	0.032307692	0.000000000
0.000000000	0.000000000	0.000000000	0.000000000
0.000000000	0.000000000	0.000177816	0.000000000
0.000000000	0.000000000	0.000381758	0.000000000
0.000000000	0.000000000	0.000453412	0.000000000
0.000000000	0.000000000	0.000199947	0.000000000
0.000000000	0.000000000	0.003136435	0.000000000
0.000000000	0.000000000	0.000000000	0.000000000
0.000000000	0.000000000	0.000000000	0.000000000

0.000000000	0.000000000	0.000000000	0.000000000
0.000000000	0.000000000	0.000000000	0.000000000
0.000000000	0.000000000	0.000451834	0.000000000
0.000000000	0.000000000	0.000000000	0.000000000
0.000000000	0.000000000	0.000000000	0.000000000
0.000000000	0.000000000	0.001285250	0.000000000
0.000000000	0.000000000	0.000000000	0.000000000
0.000000000	0.000000000	0.000098000	0.000465441
0.000000000	0.000044600	0.000280215	0.000026800
0.000000000	0.000000000	0.000000000	0.000000000
0.000000000	0.000000000	0.000000000	0.002159827

Bullous pemphigoid	Castleman disease	Celiac disease	Cicatricial pemphigoid	Cogan's syndrome
0.000000000	0.000000000	0.003115829	0.000000000	0.000000000
0.000000000	0.000592417	0.000000000	0.000000000	0.000000000
0.002642913	0.000000000	0.001296517	0.000000000	0.000000000
0.000262123	0.001822106	0.001276121	0.000000000	0.000000000
0.000119104	0.000087800	0.001426566	0.000096900	0.000199203
0.000246412	0.000144791	0.000752685	0.000000000	0.000269614
0.000000000	0.000000000	0.000000000	0.000000000	0.000000000
0.000000000	0.000000000	0.000000000	0.000000000	0.000000000
0.000000000	0.000000000	0.000159371	0.000000000	0.000000000
0.000165975	0.000000000	0.004861593	0.000000000	0.000000000
0.000000000	0.000000000	0.000000000	0.000000000	0.017513135
0.000000000	0.000000000	0.000244170	0.000000000	0.000000000
0.000000000	0.000000000	0.000000000	0.000000000	0.000000000
0.000000000	0.000000000	0.000000000	0.000000000	0.000000000
0.000000000	0.002066116	0.000383450	0.000000000	0.000000000
0.000000000	0.000000000	0.000000000	0.000000000	0.000000000
1.000000000	0.000700444	0.000716601	0.036887887	0.000000000
0.000700444	1.000000000	0.000185881	0.000000000	0.000000000
0.000716601	0.000185881	1.000000000	0.000000000	0.000000000
0.036887887	0.000000000	0.000000000	1.000000000	0.000000000
0.000000000	0.000000000	0.000000000	0.000000000	1.000000000
0.000336022	0.000000000	0.000000000	0.000000000	0.000000000
0.000000000	0.000000000	0.000081400	0.000000000	0.000000000
0.000281283	0.000295671	0.009439414	0.000088400	0.000179087
0.044462987	0.000000000	0.029248652	0.004299021	0.000000000
0.005184205	0.000000000	0.000328012	0.001318681	0.000000000
0.000000000	0.000000000	0.000000000	0.000000000	0.000000000
0.000000000	0.000154741	0.000282709	0.000000000	0.000000000
0.000234990	0.000000000	0.002876354	0.000000000	0.000000000
0.000479923	0.000000000	0.000000000	0.000000000	0.000000000
0.000790826	0.000000000	0.000351519	0.000000000	0.000000000
0.000000000	0.000000000	0.000000000	0.000000000	0.000000000
0.000327654	0.001359065	0.000409333	0.000000000	0.000000000
0.000000000	0.000000000	0.001055057	0.000000000	0.000158579
0.000000000	0.000000000	0.000121369	0.000000000	0.000000000
0.000449431	0.000952381	0.000992765	0.000000000	0.000087400
0.000786411	0.000000000	0.000081000	0.000000000	0.000000000
0.000143000	0.000432489	0.000247571	0.000530448	0.001691857
0.000321492	0.000000000	0.002249067	0.000000000	0.000000000
0.000251651	0.000296890	0.000262973	0.000000000	0.000000000
0.000602712	0.000000000	0.002693221	0.000000000	0.000000000
0.000609932	0.001497997	0.000930082	0.000075200	0.000000000
0.000482276	0.000340483	0.000225344	0.000000000	0.000941029
0.000000000	0.000000000	0.000108877	0.000000000	0.000000000
0.000000000	0.000000000	0.000360514	0.000000000	0.000847937
0.000000000	0.000000000	0.000000000	0.000000000	0.000000000

0.001283098	0.000974659	0.000111462	0.000000000	0.001151742
0.014901303	0.001128074	0.000322988	0.004042763	0.000000000
0.002078748	0.000000000	0.000150795	0.001651982	0.000000000
0.000000000	0.000000000	0.000000000	0.000000000	0.000000000
0.002880792	0.000342274	0.002369020	0.000381862	0.000181345
0.000000000	0.000000000	0.000067500	0.000000000	0.002107652
0.000000000	0.000000000	0.000000000	0.000000000	0.001766784
0.000625000	0.000716589	0.000189825	0.000000000	0.000690608
0.000000000	0.000000000	0.000000000	0.000000000	0.000000000
0.000000000	0.000000000	0.000081200	0.000000000	0.000000000
0.000544662	0.000021900	0.001519798	0.000000000	0.000056400
0.000752005	0.000929584	0.001325820	0.000000000	0.000000000
0.000000000	0.000000000	0.000100983	0.000000000	0.000000000
0.000000000	0.000000000	0.000000000	0.000000000	0.000000000
0.000290839	0.000000000	0.000523688	0.000000000	0.000000000
0.000149009	0.000266129	0.000466737	0.000072200	0.000000000
0.000000000	0.000000000	0.000000000	0.000000000	0.000000000
0.000000000	0.000000000	0.000000000	0.000000000	0.000000000
0.000000000	0.000000000	0.000000000	0.000000000	0.000000000
0.077429984	0.008615696	0.000519151	0.009328997	0.000000000
0.000119246	0.001717835	0.002188638	0.000000000	0.000149618
0.000000000	0.000000000	0.004951976	0.000000000	0.000000000
0.000000000	0.037780966	0.000000000	0.000000000	0.000000000
0.000318547	0.000000000	0.000323719	0.000000000	0.003230148
0.000000000	0.000000000	0.001759267	0.000000000	0.000000000
0.000000000	0.000000000	0.000166701	0.000000000	0.000000000
0.000000000	0.000000000	0.000334798	0.000000000	0.000000000
0.000225581	0.000310222	0.000330895	0.000000000	0.000000000
0.001767370	0.000127251	0.000740720	0.000000000	0.000153504
0.000530263	0.000000000	0.003946029	0.000000000	0.000000000
0.000189807	0.000000000	0.002603805	0.000000000	0.000000000
0.000000000	0.000000000	0.000000000	0.000000000	0.000000000
0.005279760	0.000122442	0.002443856	0.000145373	0.000000000
0.000367287	0.000000000	0.000923521	0.000000000	0.000178047
0.000000000	0.000614502	0.000116996	0.000000000	0.000000000
0.001873898	0.000000000	0.000291747	0.000662544	0.000754148
0.000000000	0.000516329	0.000210489	0.000000000	0.000000000
0.000278164	0.000000000	0.000117513	0.000000000	0.005791506
0.000000000	0.000809323	0.000074200	0.000000000	0.000572574
0.000000000	0.000000000	0.000218221	0.000000000	0.000000000
0.000639942	0.000403174	0.002150179	0.000129237	0.000158587
0.000375466	0.000889527	0.001439859	0.000152728	0.000403978
0.000000000	0.000000000	0.000000000	0.002270516	0.009137863
0.001294947	0.000129765	0.001358722	0.000104573	0.000000000
0.000937333	0.001050841	0.002483769	0.000784182	0.000519991
0.000000000	0.000000000	0.000201841	0.000000000	0.000000000
0.000000000	0.000000000	0.000000000	0.000000000	0.003773585

0.000000000	0.000000000	0.000000000	0.000000000	0.000000000
0.000000000	0.000000000	0.000155872	0.000000000	0.001809955
0.000236490	0.000828320	0.000663737	0.000000000	0.000000000
0.000000000	0.000000000	0.000000000	0.000000000	0.000000000
0.000000000	0.000000000	0.000112871	0.000000000	0.000000000
0.000640854	0.000074400	0.008222105	0.000038700	0.000136997
0.000000000	0.000000000	0.000123957	0.000000000	0.000000000
0.000249813	0.000168200	0.000866301	0.000861807	0.001234314
0.000596278	0.000321717	0.000965521	0.000079700	0.001854675
0.003103066	0.000000000	0.002449518	0.000221803	0.000000000
0.000000000	0.000000000	0.000000000	0.000000000	0.000000000

Cold agglutinin disease	CREST syndrome	Crohn's disease	Dermatitis herpetiformis	Discoid lupus
0.000000000	0.000000000	0.000332419	0.000561877	0.000401606
0.000000000	0.000000000	0.000044900	0.000000000	0.000000000
0.000000000	0.000000000	0.000571335	0.002753786	0.008078129
0.000362450	0.000149858	0.002422916	0.000250564	0.000330888
0.000000000	0.000147073	0.009084027	0.000392157	0.000729864
0.000178651	0.000349559	0.000436316	0.000000000	0.001395187
0.000000000	0.000000000	0.000000000	0.000000000	0.000000000
0.000000000	0.000000000	0.000000000	0.000000000	0.000000000
0.000000000	0.000000000	0.000395924	0.000000000	0.000000000
0.000000000	0.001793351	0.001891663	0.000611372	0.000295072
0.000000000	0.000000000	0.000089900	0.000000000	0.000000000
0.000000000	0.000000000	0.000000000	0.000000000	0.000000000
0.000000000	0.000000000	0.000000000	0.000000000	0.000000000
0.000000000	0.000000000	0.000000000	0.000000000	0.000000000
0.000000000	0.000000000	0.000666409	0.000000000	0.000000000
0.000000000	0.000000000	0.000000000	0.000000000	0.000000000
0.000336022	0.000000000	0.000281283	0.044462987	0.005184777
0.000000000	0.000000000	0.000295671	0.000000000	0.000000000
0.000000000	0.000081500	0.009439694	0.029250874	0.000328048
0.000000000	0.000000000	0.000088400	0.004299021	0.001318971
0.000000000	0.000000000	0.000179087	0.000000000	0.000000000
1.000000000	0.000000000	0.000000000	0.000000000	0.000000000
0.000000000	1.000000000	0.000000000	0.000000000	0.000000000
0.000000000	0.000000000	1.000000000	0.000357616	0.000146144
0.000000000	0.000000000	0.000357616	1.000000000	0.007909770
0.000000000	0.000000000	0.000146144	0.007909770	1.000000000
0.000000000	0.000000000	0.000000000	0.000000000	0.000000000
0.000000000	0.000000000	0.001034647	0.000000000	0.000000000
0.000000000	0.000000000	0.000846292	0.000000000	0.000000000
0.000000000	0.002739726	0.000133215	0.000000000	0.000457561
0.000000000	0.000000000	0.004270346	0.001777101	0.003530558
0.000000000	0.000000000	0.000000000	0.000000000	0.000000000
0.004712535	0.000000000	0.000000000	0.000516529	0.000000000
0.000000000	0.000000000	0.000477995	0.000193723	0.000315756
0.000000000	0.000000000	0.000132823	0.000000000	0.000000000
0.000034900	0.000069500	0.000712526	0.000416167	0.000877556
0.000000000	0.000000000	0.000044300	0.000000000	0.000000000
0.000000000	0.000435066	0.000872302	0.000000000	0.000330497
0.000000000	0.000099500	0.000581331	0.000397333	0.000217335
0.000184213	0.000000000	0.000402422	0.000000000	0.000214056
0.000000000	0.000774144	0.000636263	0.000388299	0.000000000
0.014520888	0.000113735	0.000486214	0.000657008	0.000820092
0.000000000	0.000000000	0.000424899	0.001155306	0.000000000
0.000000000	0.000000000	0.000145809	0.000000000	0.000000000
0.000000000	0.000000000	0.000137325	0.000200904	0.000000000
0.000000000	0.000000000	0.000000000	0.000000000	0.000000000

0.000000000	0.000000000	0.000675904	0.002050797	0.001943199
0.000000000	0.000000000	0.000587178	0.005810922	0.019503938
0.000000000	0.000000000	0.000085100	0.000503356	0.003018268
0.000000000	0.000000000	0.000000000	0.000000000	0.000000000
0.000138571	0.000829787	0.003842844	0.002094697	0.038727228
0.000000000	0.000000000	0.000000000	0.000000000	0.000000000
0.000000000	0.001161890	0.000108260	0.000000000	0.000000000
0.000000000	0.010053409	0.000106803	0.000000000	0.003933137
0.000000000	0.000000000	0.000000000	0.000000000	0.000000000
0.000000000	0.000000000	0.000066500	0.000000000	0.000000000
0.000000000	0.000000000	0.003681372	0.000098400	0.000185203
0.000105753	0.000104373	0.000749338	0.000737123	0.000634604
0.000000000	0.000000000	0.000039900	0.000000000	0.000000000
0.000000000	0.001320132	0.000000000	0.000000000	0.008196721
0.000379075	0.000000000	0.000061100	0.000371149	0.000000000
0.000072700	0.000029000	0.001360073	0.000223514	0.000681464
0.003781368	0.000000000	0.000000000	0.000000000	0.000000000
0.000000000	0.000000000	0.000000000	0.000000000	0.000000000
0.000000000	0.000000000	0.000000000	0.000000000	0.000000000
0.000442282	0.000000000	0.000634863	0.048029100	0.012099594
0.000198837	0.000147290	0.000813466	0.000436319	0.000472691
0.000000000	0.000000000	0.000355065	0.001658031	0.000899101
0.001748252	0.000000000	0.000000000	0.000000000	0.000000000
0.000000000	0.000000000	0.000701208	0.000678163	0.002157801
0.000000000	0.000000000	0.000089000	0.000511378	0.000703730
0.000000000	0.000000000	0.000000000	0.000000000	0.000000000
0.000000000	0.000000000	0.000000000	0.000000000	0.000000000
0.000000000	0.000736196	0.000272708	0.000000000	0.000286246
0.000000000	0.002252590	0.000527028	0.001889645	0.007185777
0.000000000	0.008506050	0.001482309	0.000820644	0.000441540
0.000000000	0.000000000	0.008006209	0.000481754	0.000000000
0.000000000	0.000000000	0.000000000	0.000885740	0.000000000
0.000000000	0.000000000	0.009198679	0.003586487	0.004018370
0.000000000	0.000000000	0.006441136	0.000283567	0.000830392
0.001326847	0.000000000	0.000043400	0.000000000	0.000000000
0.000000000	0.000000000	0.005730960	0.001460707	0.001528202
0.001757469	0.031244010	0.000163322	0.000252016	0.002781809
0.000000000	0.000000000	0.000260870	0.000403470	0.000942152
0.000000000	0.000000000	0.000168723	0.000000000	0.000000000
0.000000000	0.000000000	0.000192520	0.000186428	0.000608347
0.000050400	0.000366872	0.008457407	0.000324914	0.001085876
0.000000000	0.000246139	0.005216174	0.000485008	0.001668458
0.000000000	0.000000000	0.000796881	0.000000000	0.000000000
0.000141824	0.022014724	0.000805433	0.001194357	0.006158386
0.000230150	0.003299954	0.001389797	0.000793021	0.003565846
0.000000000	0.000000000	0.000000000	0.000714796	0.000000000
0.000000000	0.000000000	0.000000000	0.000000000	0.000000000

0.000000000	0.000000000	0.000000000	0.000000000	0.000000000
0.000000000	0.000000000	0.000997766	0.000000000	0.000000000
0.000885914	0.000097200	0.000607808	0.000172861	0.000255417
0.000000000	0.000000000	0.000044200	0.000000000	0.000000000
0.000000000	0.000000000	0.000042500	0.000333167	0.000000000
0.000000000	0.000000000	0.209365655	0.000760146	0.000239331
0.000000000	0.002811621	0.000000000	0.000000000	0.001761449
0.000000000	0.000127395	0.004777089	0.000334952	0.000450825
0.000071200	0.000248566	0.003647479	0.000555710	0.001204026
0.000000000	0.000341569	0.000763884	0.002385791	0.003517502
0.000000000	0.000000000	0.000000000	0.000000000	0.000000000

Dressler's syndrome	Endometriosis	Eosinophilic esophagitis	Eosinophilic fasciitis	Erythema nodosum
0.000000000	0.000120853	0.000000000	0.000000000	0.000235766
0.000000000	0.000000000	0.000000000	0.000000000	0.000405762
0.000000000	0.000000000	0.000000000	0.000000000	0.001281914
0.000000000	0.000307011	0.000000000	0.000089800	0.000751436
0.000000000	0.000143064	0.000000000	0.000195628	0.002886479
0.000000000	0.000200130	0.000000000	0.000173989	0.000455729
0.000000000	0.000000000	0.000000000	0.000000000	0.000000000
0.000000000	0.000000000	0.000000000	0.000000000	0.000000000
0.000000000	0.000046400	0.000000000	0.000000000	0.000000000
0.000000000	0.000000000	0.000000000	0.000000000	0.000536865
0.000000000	0.000000000	0.000000000	0.000000000	0.000000000
0.000000000	0.000000000	0.000000000	0.000000000	0.000000000
0.000000000	0.000000000	0.000000000	0.000000000	0.000000000
0.000000000	0.000000000	0.000000000	0.000000000	0.000000000
0.000000000	0.000000000	0.000379291	0.000000000	0.000000000
0.000000000	0.000000000	0.000000000	0.000000000	0.000000000
0.000000000	0.000000000	0.000235018	0.000479923	0.000790826
0.000000000	0.000154732	0.000000000	0.000000000	0.000000000
0.000000000	0.000282709	0.002876677	0.000000000	0.000351543
0.000000000	0.000000000	0.000000000	0.000000000	0.000000000
0.000000000	0.000000000	0.000000000	0.000000000	0.000000000
0.000000000	0.000000000	0.000000000	0.000000000	0.000000000
0.000000000	0.000000000	0.000000000	0.002739726	0.000000000
0.000000000	0.001034647	0.000846292	0.000133215	0.004270346
0.000000000	0.000000000	0.000000000	0.000000000	0.001777101
0.000000000	0.000000000	0.000000000	0.000457561	0.003530558
1.000000000	0.000000000	0.000000000	0.000000000	0.000000000
0.000000000	1.000000000	0.000000000	0.000000000	0.000000000
0.000000000	0.000000000	1.000000000	0.000000000	0.000000000
0.000000000	0.000000000	0.000000000	1.000000000	0.000371609
0.000000000	0.000000000	0.000000000	0.000371609	1.000000000
0.000000000	0.000000000	0.000000000	0.000000000	0.000000000
0.000000000	0.000000000	0.000000000	0.000000000	0.000000000
0.000000000	0.001352330	0.000000000	0.000000000	0.000474975
0.000000000	0.000000000	0.000000000	0.000000000	0.000000000
0.000000000	0.000046500	0.000000000	0.000086800	0.000912008
0.000000000	0.000000000	0.000000000	0.000000000	0.000000000
0.000000000	0.000000000	0.001131025	0.000540950	0.000686551
0.000000000	0.000123378	0.000000000	0.000099300	0.000249833
0.000000000	0.000000000	0.000000000	0.000000000	0.000199654
0.000000000	0.000059300	0.000267451	0.000574933	0.000220046
0.000271528	0.000072800	0.000000000	0.000113520	0.000528332
0.000000000	0.000000000	0.000000000	0.000000000	0.001753203
0.000000000	0.003469897	0.000000000	0.000000000	0.000000000
0.000000000	0.000000000	0.000000000	0.000000000	0.000530363
0.000000000	0.000000000	0.000000000	0.000000000	0.000000000

0.000000000	0.000061900	0.000000000	0.000000000	0.009157509
0.000000000	0.000076000	0.000377444	0.000364000	0.002698327
0.000000000	0.000093900	0.000000000	0.004360465	0.000682967
0.000000000	0.000000000	0.000000000	0.000000000	0.000000000
0.000000000	0.000448756	0.000000000	0.000286926	0.002290974
0.000000000	0.000000000	0.000000000	0.000000000	0.000000000
0.000000000	0.000000000	0.000000000	0.000000000	0.000000000
0.000000000	0.000000000	0.000000000	0.003072197	0.000981905
0.000000000	0.000000000	0.000000000	0.000000000	0.000000000
0.000000000	0.000000000	0.000000000	0.000000000	0.000000000
0.000000000	0.000238460	0.000021900	0.000056200	0.000215522
0.000000000	0.000041900	0.000000000	0.000000000	0.000259977
0.000000000	0.000000000	0.000000000	0.000000000	0.000000000
0.000000000	0.000000000	0.000000000	0.000000000	0.000000000
0.000000000	0.000000000	0.000000000	0.000000000	0.000000000
0.000000000	0.000061500	0.000000000	0.000028900	0.000287900
0.000000000	0.000063100	0.000000000	0.000621504	0.000000000
0.000000000	0.000000000	0.000000000	0.000000000	0.000000000
0.000000000	0.000000000	0.000000000	0.000000000	0.000000000
0.000000000	0.000074800	0.000216357	0.000344649	0.001164069
0.000000000	0.000061300	0.000000000	0.000195915	0.000947711
0.000000000	0.000000000	0.000000000	0.000000000	0.000272084
0.000000000	0.000000000	0.000000000	0.000000000	0.000000000
0.000000000	0.000055100	0.000000000	0.000255787	0.006542700
0.000000000	0.000066900	0.000000000	0.000000000	0.000000000
0.000000000	0.000000000	0.000000000	0.000000000	0.000000000
0.000000000	0.000000000	0.000000000	0.000000000	0.000000000
0.000000000	0.000000000	0.000000000	0.000727273	0.000875876
0.000000000	0.000047700	0.000000000	0.001345291	0.001449780
0.000000000	0.000000000	0.000000000	0.000000000	0.000486500
0.000000000	0.000000000	0.000000000	0.000000000	0.002695977
0.000000000	0.000068100	0.000000000	0.000000000	0.000000000
0.000000000	0.000191982	0.000035000	0.000145858	0.001756960
0.000000000	0.000049900	0.000000000	0.000431146	0.000711422
0.000000000	0.000000000	0.000000000	0.000000000	0.000000000
0.000000000	0.000000000	0.000000000	0.000460193	0.023027135
0.000000000	0.000000000	0.000000000	0.005928122	0.000430246
0.000000000	0.000000000	0.000000000	0.000000000	0.002060548
0.000000000	0.000185420	0.000000000	0.000000000	0.000000000
0.000000000	0.000000000	0.000000000	0.000000000	0.003448672
0.000000000	0.000358087	0.000035400	0.000201314	0.000924027
0.000000000	0.000114649	0.000000000	0.000215034	0.019619367
0.000000000	0.000000000	0.000000000	0.000000000	0.002959455
0.000000000	0.000052600	0.000195052	0.009382856	0.000927386
0.000000000	0.000238219	0.000000000	0.000565803	0.001021688
0.000000000	0.000000000	0.000000000	0.000000000	0.000000000
0.000000000	0.000000000	0.000000000	0.000000000	0.000000000

0.000000000	0.000000000	0.000000000	0.000000000	0.000000000
0.000000000	0.000000000	0.000000000	0.000000000	0.002486016
0.000000000	0.000284970	0.000000000	0.000145398	0.000408213
0.000000000	0.000000000	0.000000000	0.000000000	0.000000000
0.000000000	0.000000000	0.000000000	0.000000000	0.000000000
0.000000000	0.000450338	0.000372377	0.000000000	0.003536068
0.000000000	0.000000000	0.000000000	0.000000000	0.000000000
0.000000000	0.000059000	0.000000000	0.000000000	0.011645617
0.000000000	0.000233716	0.000034800	0.000212955	0.006395981
0.000000000	0.000080300	0.000000000	0.000792842	0.001102970
0.000000000	0.000000000	0.000000000	0.000000000	0.000000000

Essential mixed cryoglobulinemia	Evans syndrome	Fibromyalgia	Fibrosing alveolitis	Glomerulonephritis
0.000000000	0.000000000	0.000311023	0.000000000	0.000197769
0.000000000	0.000000000	0.000000000	0.000000000	0.000070000
0.000000000	0.000000000	0.000000000	0.000000000	0.000000000
0.000000000	0.000060100	0.000178261	0.000059600	0.011048850
0.000000000	0.000000000	0.005766686	0.000194363	0.001190570
0.000181719	0.001676964	0.000479261	0.000430108	0.003653918
0.000000000	0.000000000	0.000000000	0.000000000	0.000000000
0.000000000	0.000000000	0.000000000	0.000000000	0.000000000
0.000000000	0.000000000	0.000076800	0.000000000	0.000226501
0.000000000	0.000978063	0.000159787	0.000268709	0.000821044
0.000000000	0.000000000	0.000000000	0.000000000	0.000052600
0.000000000	0.000000000	0.000000000	0.000000000	0.000052100
0.000000000	0.000000000	0.000000000	0.000000000	0.000000000
0.000000000	0.000000000	0.000000000	0.000000000	0.000035100
0.000000000	0.000000000	0.000000000	0.000000000	0.000253910
0.000000000	0.000000000	0.000000000	0.000000000	0.000000000
0.000000000	0.000327654	0.000000000	0.000000000	0.000449438
0.000000000	0.001359065	0.000000000	0.000000000	0.000952397
0.000000000	0.000409366	0.001055116	0.000121379	0.000992802
0.000000000	0.000000000	0.000000000	0.000000000	0.000000000
0.000000000	0.000000000	0.000158579	0.000000000	0.000087400
0.000000000	0.004712535	0.000000000	0.000000000	0.000034900
0.000000000	0.000000000	0.000000000	0.000000000	0.000069500
0.000000000	0.000000000	0.000477995	0.000132823	0.000712526
0.000000000	0.000516529	0.000193723	0.000000000	0.000416167
0.000000000	0.000000000	0.000315756	0.000000000	0.000877556
0.000000000	0.000000000	0.000000000	0.000000000	0.000000000
0.000000000	0.000000000	0.001352330	0.000000000	0.000046500
0.000000000	0.000000000	0.000000000	0.000000000	0.000000000
0.000000000	0.000000000	0.000000000	0.000000000	0.000086800
0.000000000	0.000000000	0.000474975	0.000000000	0.000912008
1.000000000	0.000000000	0.000000000	0.000000000	0.001016795
0.000000000	1.000000000	0.000000000	0.000000000	0.000069600
0.000000000	0.000000000	1.000000000	0.000000000	0.000072400
0.000000000	0.000000000	0.000000000	1.000000000	0.000103957
0.001016795	0.000069600	0.000072400	0.000103957	1.000000000
0.000000000	0.000000000	0.000000000	0.001140251	0.013294463
0.000000000	0.000000000	0.000193134	0.000213242	0.013183813
0.000000000	0.000400080	0.000126522	0.000000000	0.001195230
0.000000000	0.000454298	0.000176819	0.000000000	0.000863404
0.000000000	0.001184133	0.000359669	0.000000000	0.000244774
0.000308487	0.018642688	0.000079200	0.000225929	0.003966950
0.001958864	0.000000000	0.000000000	0.000000000	0.009138138
0.000000000	0.000000000	0.008153720	0.000000000	0.000115605
0.000000000	0.000411072	0.000000000	0.000000000	0.000330693
0.000000000	0.000000000	0.000000000	0.000000000	0.000000000

0.003889886	0.000000000	0.000261318	0.000000000	0.003164239
0.000190440	0.000000000	0.000133589	0.000000000	0.000492265
0.000000000	0.000000000	0.000134048	0.000000000	0.000000000
0.000000000	0.000000000	0.000000000	0.000000000	0.000000000
0.000160215	0.000883947	0.003478129	0.000265291	0.075604918
0.000000000	0.000000000	0.000221963	0.000000000	0.000000000
0.000932836	0.000000000	0.000000000	0.000000000	0.007307283
0.000000000	0.000000000	0.000678794	0.001768868	0.001146982
0.000000000	0.000000000	0.000000000	0.000000000	0.000000000
0.000000000	0.000000000	0.000000000	0.000000000	0.000000000
0.000000000	0.000022500	0.001376051	0.000033700	0.000759008
0.000000000	0.000157365	0.000195529	0.000103386	0.001636073
0.000000000	0.000000000	0.001216343	0.000000000	0.000000000
0.000000000	0.000000000	0.000000000	0.000000000	0.000399077
0.000000000	0.000000000	0.000000000	0.000000000	0.000048700
0.000000000	0.001074473	0.000086900	0.000000000	0.002233751
0.000000000	0.001629726	0.000000000	0.000000000	0.000337331
0.000000000	0.000000000	0.000000000	0.000000000	0.000000000
0.000000000	0.000000000	0.000000000	0.000000000	0.000000000
0.000000000	0.000174505	0.000000000	0.000000000	0.000398983
0.000552181	0.000000000	0.002073320	0.000145978	0.001374256
0.000000000	0.000000000	0.000000000	0.000000000	0.000253040
0.000000000	0.000000000	0.000000000	0.000000000	0.000447643
0.001092299	0.000000000	0.000362845	0.000251509	0.007964672
0.000000000	0.000000000	0.000000000	0.000000000	0.000052200
0.000000000	0.000000000	0.000000000	0.000000000	0.000000000
0.000000000	0.000000000	0.000000000	0.000000000	0.000035100
0.000549904	0.000000000	0.004699672	0.000000000	0.000232547
0.000000000	0.000377472	0.001247736	0.001480275	0.001498926
0.000000000	0.000480943	0.000300903	0.000930990	0.000837170
0.000000000	0.000000000	0.000115774	0.000000000	0.000307100
0.000000000	0.000000000	0.000000000	0.000000000	0.000000000
0.000000000	0.000036600	0.001283038	0.000054600	0.001218302
0.000000000	0.000000000	0.004920103	0.000000000	0.000443387
0.000000000	0.006663890	0.000000000	0.000000000	0.000221688
0.000000000	0.000000000	0.000000000	0.000000000	0.000198590
0.000607533	0.000000000	0.001718111	0.001079719	0.000943673
0.000000000	0.000000000	0.000143606	0.000000000	0.000820611
0.000000000	0.000000000	0.000000000	0.000000000	0.000667646
0.000000000	0.000000000	0.000718649	0.000000000	0.007388023
0.000086600	0.000057600	0.007162873	0.000380764	0.004745679
0.000000000	0.000061700	0.000386179	0.003406789	0.002261164
0.000000000	0.000000000	0.000417275	0.000000000	0.000525727
0.000000000	0.000105790	0.001477697	0.003046859	0.002999585
0.000640130	0.000000000	0.003855422	0.001180040	0.004383815
0.000000000	0.000000000	0.000000000	0.000000000	0.000000000
0.000000000	0.000000000	0.000000000	0.000000000	0.000000000

0.000000000	0.000000000	0.000000000	0.000000000	0.000000000
0.000000000	0.000000000	0.000142248	0.000000000	0.000494501
0.000099300	0.012802768	0.000062300	0.000000000	0.003268871
0.000000000	0.000000000	0.000000000	0.000000000	0.000000000
0.000000000	0.000000000	0.000000000	0.000000000	0.000184629
0.000000000	0.000077900	0.000286018	0.000232599	0.001088919
0.000000000	0.000000000	0.000635274	0.002400000	0.000104882
0.000000000	0.000102166	0.000512235	0.000152168	0.001732629
0.000731144	0.000088800	0.000765450	0.000203839	0.030559608
0.000000000	0.000345383	0.000295406	0.000000000	0.000277303
0.000000000	0.000000000	0.000000000	0.000000000	0.000000000

Goodpasture's syndrome	Granulomatosis with Polyangiitis	Graves disease	Guillain-Barre syndrome
0.000423549	0.000243013	0.005710083	0.000491194
0.000000000	0.000000000	0.000000000	0.000000000
0.000000000	0.000000000	0.001119867	0.000132066
0.000149147	0.000680851	0.000134489	0.000627382
0.000194590	0.001101126	0.000614392	0.000232659
0.000344768	0.001666840	0.000730630	0.000804810
0.000000000	0.000000000	0.000000000	0.000000000
0.000000000	0.000000000	0.000000000	0.000000000
0.000000000	0.000000000	0.000000000	0.001115795
0.000000000	0.000465487	0.001582584	0.000413028
0.000000000	0.000000000	0.000000000	0.000000000
0.000000000	0.000000000	0.000248287	0.000000000
0.000000000	0.000000000	0.000000000	0.000000000
0.000000000	0.000000000	0.000000000	0.000000000
0.000000000	0.000279148	0.000138562	0.000000000
0.000000000	0.000000000	0.000000000	0.000000000
0.000786411	0.000143010	0.000321505	0.000251667
0.000000000	0.000432526	0.000000000	0.000296912
0.000081000	0.000247594	0.002249223	0.000262996
0.000000000	0.000530504	0.000000000	0.000000000
0.000000000	0.001692047	0.000000000	0.000000000
0.000000000	0.000000000	0.000000000	0.000184230
0.000000000	0.000434452	0.000099400	0.000000000
0.000044300	0.000872302	0.000581331	0.000402422
0.000000000	0.000000000	0.000397333	0.000000000
0.000000000	0.000330497	0.000217335	0.000214056
0.000000000	0.000000000	0.000000000	0.000000000
0.000000000	0.000000000	0.000123378	0.000000000
0.000000000	0.001131025	0.000000000	0.000000000
0.000000000	0.000540950	0.000099300	0.000000000
0.000000000	0.000686551	0.000249833	0.000199654
0.000000000	0.000000000	0.000000000	0.000000000
0.000000000	0.000000000	0.000400080	0.000454298
0.000000000	0.000193134	0.000126522	0.000176819
0.001140251	0.000213242	0.000000000	0.000000000
0.013294463	0.013183813	0.001195230	0.000863404
1.000000000	0.004940393	0.000148090	0.000355114
0.004940393	1.000000000	0.000753634	0.000370645
0.000148090	0.000753634	1.000000000	0.000402887
0.000355114	0.000370645	0.000402887	1.000000000
0.000000000	0.000231624	0.061768502	0.000471095
0.000188437	0.000322080	0.001066193	0.001110032
0.002186390	0.005520926	0.000225317	0.000303053
0.000000000	0.000000000	0.000000000	0.000000000
0.000000000	0.002773925	0.000191703	0.002404269
0.000000000	0.000000000	0.000000000	0.001323877

0.000506201	0.008888114	0.001202405	0.000222453
0.000000000	0.000213630	0.000270060	0.000096900
0.000000000	0.000000000	0.000000000	0.000000000
0.000000000	0.000000000	0.000000000	0.000000000
0.000775243	0.004764253	0.002836892	0.001466870
0.000000000	0.000000000	0.000000000	0.000000000
0.005496519	0.092000000	0.000188041	0.000000000
0.000889944	0.002185792	0.000319649	0.000000000
0.000000000	0.000000000	0.000000000	0.000000000
0.000000000	0.000000000	0.000000000	0.015952144
0.000000000	0.000320728	0.001368193	0.005230104
0.000880555	0.000408284	0.006969383	0.013517784
0.000000000	0.000000000	0.000079100	0.002030769
0.000000000	0.000000000	0.000000000	0.000000000
0.000000000	0.000300458	0.000206492	0.002501316
0.000057800	0.000755474	0.001918575	0.000241346
0.000000000	0.000000000	0.000000000	0.000233118
0.000000000	0.000000000	0.000000000	0.000000000
0.000000000	0.000000000	0.000000000	0.000470588
0.000512251	0.000620572	0.000595376	0.000470544
0.000000000	0.002491990	0.000128018	0.009838818
0.000275292	0.000134273	0.002408983	0.000238010
0.000000000	0.000000000	0.000000000	0.002490981
0.002655203	0.041987544	0.000491512	0.000629939
0.000000000	0.000000000	0.003102482	0.000000000
0.000000000	0.000000000	0.000716259	0.000000000
0.000000000	0.000000000	0.000565465	0.000000000
0.000000000	0.002449738	0.000307530	0.000362976
0.000370316	0.003417992	0.001031057	0.003351761
0.000816803	0.000308604	0.001514704	0.000110387
0.000000000	0.000591279	0.000204625	0.000000000
0.000000000	0.000000000	0.000000000	0.000000000
0.000054600	0.000271661	0.000857913	0.000217074
0.000000000	0.000620829	0.000529328	0.000094100
0.000000000	0.000000000	0.000000000	0.000000000
0.000000000	0.003903654	0.000565808	0.000000000
0.000000000	0.000607857	0.000166203	0.000132608
0.000000000	0.005525952	0.000189681	0.000000000
0.000000000	0.001121850	0.000088900	0.000000000
0.000000000	0.000656907	0.000310385	0.000301283
0.000193971	0.002272727	0.001771699	0.000416930
0.000275566	0.007525376	0.001153651	0.001138358
0.000000000	0.015064836	0.000464296	0.000000000
0.000314597	0.003238306	0.001019087	0.000157249
0.000168634	0.003513065	0.002373134	0.001171904
0.000000000	0.000000000	0.000295145	0.002384316
0.000000000	0.000000000	0.000000000	0.000000000

0.000000000	0.000000000	0.000147218	0.000000000
0.001116487	0.004183693	0.000094200	0.000000000
0.001061622	0.000811202	0.001964186	0.003644194
0.000000000	0.001591512	0.012442144	0.000000000
0.000000000	0.000000000	0.000000000	0.012000000
0.000038800	0.000912841	0.000742210	0.000327284
0.000000000	0.000000000	0.000101209	0.000000000
0.000050700	0.003284352	0.001140290	0.000510861
0.001242313	0.071457796	0.001753891	0.001210783
0.000000000	0.000000000	0.003945782	0.000216321
0.000000000	0.000000000	0.000099100	0.000267953

Hashimoto's thyroiditis	Hemolytic anemia	Henoch-Schonlein purpura	Interstitial cystitis	Kawasaki disease
0.009828904	0.000169348	0.000300436	0.000000000	0.000000000
0.000000000	0.000077000	0.000000000	0.000000000	0.000000000
0.002940216	0.000131518	0.000795650	0.000000000	0.000349864
0.000215523	0.000411593	0.000592501	0.000109938	0.000254168
0.000331263	0.000529322	0.000355761	0.000000000	0.000303295
0.000855770	0.004967984	0.001034967	0.000000000	0.000918748
0.000000000	0.000000000	0.000000000	0.000000000	0.000000000
0.000000000	0.000000000	0.000000000	0.000000000	0.000000000
0.000000000	0.000177260	0.000000000	0.000000000	0.000000000
0.002456332	0.001958712	0.000213812	0.000000000	0.000377615
0.000429830	0.000000000	0.000000000	0.000000000	0.000000000
0.000575705	0.000000000	0.000000000	0.000000000	0.000000000
0.000000000	0.000000000	0.000000000	0.000000000	0.000000000
0.000000000	0.000000000	0.000000000	0.000000000	0.000000000
0.000446362	0.000214854	0.000000000	0.000000000	0.000000000
0.000000000	0.000000000	0.000000000	0.000000000	0.000000000
0.000602712	0.000609952	0.000482276	0.000000000	0.000000000
0.000000000	0.001498049	0.000000000	0.000000000	0.000000000
0.002693412	0.000930139	0.000225361	0.000108889	0.000360538
0.000000000	0.000075200	0.000000000	0.000000000	0.000000000
0.000000000	0.000000000	0.000941029	0.000000000	0.000847937
0.000000000	0.014521452	0.000000000	0.000000000	0.000000000
0.000772947	0.000113615	0.000000000	0.000000000	0.000000000
0.000636263	0.000486214	0.000424899	0.000145809	0.000137325
0.000388299	0.000657008	0.001155306	0.000000000	0.000200904
0.000000000	0.000820092	0.000000000	0.000000000	0.000000000
0.000000000	0.000271528	0.000000000	0.000000000	0.000000000
0.000059300	0.000072800	0.000000000	0.003469897	0.000000000
0.000267451	0.000000000	0.000000000	0.000000000	0.000000000
0.000574933	0.000113520	0.000000000	0.000000000	0.000000000
0.000220046	0.000528332	0.001753203	0.000000000	0.000530363
0.000000000	0.000308487	0.001958864	0.000000000	0.000000000
0.001184133	0.018642688	0.000000000	0.000000000	0.000411072
0.000359669	0.000079200	0.000000000	0.008153720	0.000000000
0.000000000	0.000225929	0.000000000	0.000000000	0.000000000
0.000244774	0.003966950	0.009138138	0.000115605	0.000330693
0.000000000	0.000188437	0.002186390	0.000000000	0.000000000
0.000231624	0.000322080	0.005520926	0.000000000	0.002773925
0.061768502	0.001066193	0.000225317	0.000000000	0.000191703
0.000471095	0.001110032	0.000303053	0.000000000	0.002404269
1.000000000	0.000929954	0.000275368	0.000000000	0.000179308
0.000929954	1.000000000	0.000316178	0.000102055	0.000834931
0.000275368	0.000316178	1.000000000	0.000000000	0.010646226
0.000000000	0.000102055	0.000000000	1.000000000	0.000000000
0.000179308	0.000834931	0.010646226	0.000000000	1.000000000
0.000000000	0.000000000	0.000000000	0.000000000	0.000000000

0.000000000	0.000313033	0.042123894	0.000000000	0.000613936
0.000748860	0.000139260	0.000153421	0.000000000	0.000177242
0.000279330	0.000000000	0.000000000	0.000000000	0.000000000
0.000000000	0.000000000	0.000000000	0.000000000	0.000000000
0.001658596	0.009283673	0.001989956	0.000721441	0.001180638
0.000000000	0.000000000	0.000000000	0.000000000	0.000000000
0.000315308	0.000145127	0.013200606	0.000000000	0.004448607
0.001291990	0.000922968	0.000942152	0.000000000	0.000543183
0.000000000	0.000000000	0.000000000	0.000000000	0.000000000
0.000569909	0.000113272	0.000000000	0.000000000	0.000933956
0.000669583	0.000579904	0.000033000	0.000206744	0.000158099
0.002755785	0.002020156	0.000000000	0.000000000	0.000835389
0.000000000	0.000000000	0.000000000	0.000000000	0.000159058
0.000379147	0.000226483	0.000000000	0.000000000	0.000799361
0.000000000	0.000196702	0.000000000	0.000000000	0.000000000
0.000137378	0.008040912	0.000140590	0.000041600	0.000146616
0.000000000	0.011461627	0.000379867	0.000000000	0.000000000
0.000000000	0.000000000	0.000000000	0.000000000	0.000000000
0.000000000	0.000000000	0.000000000	0.000000000	0.000000000
0.000980072	0.000986166	0.000146466	0.000000000	0.000113941
0.000000000	0.000485330	0.000311651	0.000171013	0.000000000
0.003508772	0.006492692	0.000000000	0.000296209	0.000152660
0.000000000	0.000185563	0.000000000	0.000000000	0.000000000
0.000433802	0.001375852	0.013662044	0.000000000	0.009089576
0.009672325	0.000000000	0.000000000	0.000000000	0.000000000
0.003468459	0.000000000	0.000000000	0.000000000	0.000000000
0.001546620	0.000000000	0.000000000	0.000000000	0.000000000
0.000510595	0.000516458	0.000323992	0.000000000	0.000496623
0.001639344	0.001384680	0.000712712	0.000000000	0.001294130
0.003130148	0.001469265	0.000190241	0.000000000	0.000000000
0.000631380	0.000455789	0.000000000	0.000000000	0.000000000
0.000000000	0.000000000	0.000000000	0.000000000	0.000000000
0.000649262	0.000564320	0.000298843	0.000069200	0.000526584
0.000261301	0.000191644	0.000439786	0.000000000	0.000113993
0.001116962	0.005595787	0.000000000	0.000000000	0.000000000
0.000000000	0.000307482	0.000469337	0.000000000	0.000388727
0.000876040	0.001022090	0.000267559	0.000238067	0.000000000
0.000487171	0.000255708	0.000890076	0.000000000	0.000000000
0.001587302	0.000069500	0.000507958	0.000292697	0.000000000
0.000289151	0.000545497	0.001152664	0.000000000	0.004318988
0.001114499	0.001838235	0.000276357	0.000168939	0.000406894
0.000827815	0.001184194	0.000202312	0.000000000	0.000389701
0.000000000	0.000000000	0.000000000	0.000000000	0.000225963
0.001523206	0.002307000	0.000458400	0.000190627	0.000551764
0.005735239	0.002446426	0.000608550	0.001357313	0.000635189
0.000370370	0.000000000	0.000000000	0.000000000	0.000392824
0.000000000	0.000000000	0.000000000	0.000000000	0.000000000

0.000000000	0.000075000	0.000000000	0.000000000	0.000000000
0.000000000	0.000000000	0.003686836	0.000000000	0.002586106
0.001110243	0.045018806	0.001059182	0.000084700	0.001959897
0.003524393	0.000000000	0.000000000	0.000000000	0.000000000
0.000000000	0.000422297	0.000000000	0.000000000	0.000000000
0.000471407	0.002190624	0.000392553	0.000146964	0.000104508
0.000000000	0.000191968	0.000631912	0.000000000	0.000000000
0.000441922	0.000498427	0.000508118	0.000047300	0.000973301
0.000326472	0.002366562	0.021648760	0.000077800	0.048172552
0.006256015	0.000978242	0.000459643	0.000000000	0.000270636
0.000381098	0.000000000	0.000000000	0.000000000	0.000000000

Lambert-Eaton syndrome	Leukocytoclastic vasculitis	Lichen planus	Lichen sclerosus	Ligneous conjunctivitis
0.000000000	0.000288060	0.000354862	0.000000000	0.000000000
0.000000000	0.000000000	0.000000000	0.000000000	0.000000000
0.000000000	0.000766577	0.011476406	0.002694328	0.000000000
0.000000000	0.000363769	0.002078954	0.000141423	0.000121788
0.000000000	0.000570977	0.000233934	0.000000000	0.000000000
0.000000000	0.001158497	0.000286123	0.000000000	0.000000000
0.000000000	0.000000000	0.000000000	0.000000000	0.000000000
0.000000000	0.000000000	0.000000000	0.000000000	0.000000000
0.000000000	0.000000000	0.000000000	0.000000000	0.000000000
0.000000000	0.000622665	0.001311945	0.000000000	0.000000000
0.000000000	0.000000000	0.000000000	0.000000000	0.000000000
0.000000000	0.000000000	0.000000000	0.000000000	0.000000000
0.000000000	0.000000000	0.000000000	0.000000000	0.000000000
0.000000000	0.000000000	0.000000000	0.000000000	0.000000000
0.000000000	0.000561377	0.000000000	0.000000000	0.000000000
0.000000000	0.000000000	0.000000000	0.000000000	0.000000000
0.000000000	0.001283098	0.014901303	0.002079002	0.000000000
0.000000000	0.000974659	0.001128159	0.000000000	0.000000000
0.000000000	0.000111470	0.000323016	0.000150818	0.000000000
0.000000000	0.000000000	0.004042763	0.001652437	0.000000000
0.000000000	0.001151742	0.000000000	0.000000000	0.000000000
0.000000000	0.000000000	0.000000000	0.000000000	0.000000000
0.000000000	0.000000000	0.000000000	0.000000000	0.000000000
0.000000000	0.000675904	0.000587231	0.000085100	0.000000000
0.000000000	0.002050797	0.005810922	0.000503356	0.000000000
0.000000000	0.001943199	0.019503938	0.003018268	0.000000000
0.000000000	0.000000000	0.000000000	0.000000000	0.000000000
0.000000000	0.000061900	0.000076000	0.000093900	0.000000000
0.000000000	0.000000000	0.000377444	0.000000000	0.000000000
0.000000000	0.000000000	0.000364000	0.004360465	0.000000000
0.000000000	0.009157509	0.002698509	0.000683060	0.000000000
0.000000000	0.003889886	0.000190440	0.000000000	0.000000000
0.000000000	0.000000000	0.000000000	0.000000000	0.000000000
0.000000000	0.000261318	0.000133589	0.000134048	0.000000000
0.000000000	0.000000000	0.000000000	0.000000000	0.000000000
0.000000000	0.003164239	0.000492280	0.000000000	0.000000000
0.000000000	0.000506201	0.000000000	0.000000000	0.000000000
0.000000000	0.008888114	0.000213641	0.000000000	0.000000000
0.000000000	0.001202405	0.000270060	0.000000000	0.000000000
0.001323877	0.000222453	0.000096900	0.000000000	0.000000000
0.000000000	0.000000000	0.000748860	0.000279330	0.000000000
0.000000000	0.000313033	0.000139260	0.000000000	0.000000000
0.000000000	0.042123894	0.000153433	0.000000000	0.000000000
0.000000000	0.000000000	0.000000000	0.000000000	0.000000000
0.000000000	0.000613936	0.000177253	0.000000000	0.000000000
1.000000000	0.000000000	0.000000000	0.000000000	0.000000000

0.000000000	1.000000000	0.002558507	0.000345841	0.000000000
0.000000000	0.002558507	1.000000000	0.024881141	0.000000000
0.000000000	0.000345841	0.024881141	1.000000000	0.000000000
0.000000000	0.000000000	0.000000000	0.000000000	1.000000000
0.000000000	0.002421408	0.006283994	0.000531150	0.000000000
0.000000000	0.000000000	0.000000000	0.000000000	0.000000000
0.000000000	0.008937640	0.000165098	0.000000000	0.000000000
0.000000000	0.001789869	0.000235423	0.000000000	0.000000000
0.000000000	0.000629327	0.000000000	0.000000000	0.000000000
0.002030457	0.000000000	0.000000000	0.000000000	0.000000000
0.000101847	0.000065700	0.000162580	0.000000000	0.000000000
0.003174603	0.000000000	0.000941784	0.000000000	0.000000000
0.000000000	0.000000000	0.000000000	0.000000000	0.000000000
0.000000000	0.000000000	0.000000000	0.000000000	0.000000000
0.000599401	0.000252908	0.000000000	0.000000000	0.000000000
0.000000000	0.000770643	0.000089100	0.000000000	0.000000000
0.000000000	0.000000000	0.000000000	0.000000000	0.000000000
0.000000000	0.000000000	0.000000000	0.000000000	0.000000000
0.000000000	0.000000000	0.000000000	0.000000000	0.000000000
0.000000000	0.001292732	0.021157105	0.001329984	0.000000000
0.000100761	0.001011879	0.000234145	0.000000000	0.000000000
0.000000000	0.000000000	0.000420850	0.000439754	0.000000000
0.000000000	0.000000000	0.000000000	0.000000000	0.000000000
0.000000000	0.014792604	0.001157207	0.000000000	0.000000000
0.000000000	0.000000000	0.000000000	0.000000000	0.000000000
0.000000000	0.000000000	0.000000000	0.000000000	0.000000000
0.000000000	0.000000000	0.000000000	0.000000000	0.000000000
0.000000000	0.000464612	0.000000000	0.000000000	0.000000000
0.000780153	0.000954502	0.001269091	0.000391287	0.000000000
0.000000000	0.000463177	0.001841826	0.000480538	0.000000000
0.000000000	0.000000000	0.000458295	0.000000000	0.000000000
0.000000000	0.000000000	0.000193013	0.000000000	0.000000000
0.000000000	0.000857558	0.014554916	0.002170269	0.000000000
0.000000000	0.000574465	0.000664452	0.000000000	0.000000000
0.000000000	0.000000000	0.000581347	0.000000000	0.000000000
0.000000000	0.008758683	0.003045685	0.000795545	0.000000000
0.000000000	0.001419172	0.000335368	0.000271370	0.000000000
0.000000000	0.001885212	0.000000000	0.000000000	0.000000000
0.000000000	0.000322997	0.000000000	0.000000000	0.000000000
0.000000000	0.000251335	0.000000000	0.000000000	0.000000000
0.000000000	0.000863265	0.000552129	0.000028400	0.000000000
0.000000000	0.000659612	0.001810904	0.000376932	0.000000000
0.000000000	0.000955292	0.000161434	0.000000000	0.000000000
0.000000000	0.000551536	0.004441624	0.007210902	0.000000000
0.000116863	0.002253267	0.003842743	0.000101916	0.000000000
0.003577818	0.000000000	0.000000000	0.000000000	0.000000000
0.000000000	0.000000000	0.000000000	0.000000000	0.000000000

0.000000000	0.000000000	0.000000000	0.000000000	0.000000000
0.000000000	0.000000000	0.000000000	0.000000000	0.000000000
0.000000000	0.000784280	0.000564671	0.000000000	0.000000000
0.000000000	0.000000000	0.000000000	0.000000000	0.000000000
0.000000000	0.000686577	0.000000000	0.000000000	0.000000000
0.000000000	0.000613725	0.000541658	0.000037400	0.000000000
0.000000000	0.000000000	0.000000000	0.000000000	0.000000000
0.000000000	0.001394968	0.001046390	0.000048500	0.000000000
0.000000000	0.027597417	0.001442209	0.000122109	0.000000000
0.000000000	0.000447868	0.010701087	0.006349206	0.000000000
0.000000000	0.000000000	0.000000000	0.000000000	0.000000000

Lupus	Meniere's disease	Microscopic polyangiitis	Mixed connective tissue disease
0.000833882	0.000000000	0.000348857	0.000000000
0.000149496	0.000000000	0.000000000	0.001068757
0.002001859	0.000000000	0.000000000	0.000275824
0.003511706	0.000000000	0.000173742	0.000284414
0.008600385	0.000117735	0.000092700	0.002392993
0.054018205	0.000121007	0.001031419	0.004702313
0.000000000	0.000000000	0.000000000	0.000000000
0.000000000	0.000000000	0.000000000	0.000000000
0.001495006	0.000000000	0.000000000	0.000000000
0.003636327	0.000000000	0.000712082	0.002655455
0.000074800	0.003860356	0.000000000	0.000000000
0.000106272	0.000000000	0.000000000	0.000000000
0.000053500	0.000000000	0.000000000	0.000000000
0.000021400	0.000000000	0.000000000	0.000000000
0.000209209	0.000000000	0.000000000	0.000000000
0.000042800	0.000000000	0.000000000	0.000000000
0.002881172	0.000000000	0.000000000	0.000625078
0.000342324	0.000000000	0.000000000	0.000717103
0.002369344	0.000067500	0.000000000	0.000189890
0.000381914	0.000000000	0.000000000	0.000000000
0.000181370	0.002108336	0.001767565	0.000690846
0.000138590	0.000000000	0.000000000	0.000000000
0.000828518	0.000000000	0.001160542	0.010043942
0.003843209	0.000000000	0.000108314	0.000106858
0.002094871	0.000000000	0.000000000	0.000000000
0.038730546	0.000000000	0.000000000	0.003935071
0.000000000	0.000000000	0.000000000	0.000000000
0.000448796	0.000000000	0.000000000	0.000000000
0.000000000	0.000000000	0.000000000	0.000000000
0.000286950	0.000000000	0.000000000	0.003073141
0.002291185	0.000000000	0.000000000	0.000982180
0.000160229	0.000000000	0.000932836	0.000000000
0.000884023	0.000000000	0.000000000	0.000000000
0.003478393	0.000222111	0.000000000	0.000679302
0.000265314	0.000000000	0.000000000	0.001769390
0.075610333	0.000000000	0.007309039	0.001147273
0.000775309	0.000000000	0.005496519	0.000890208
0.004764676	0.000000000	0.092048447	0.002186987
0.002837094	0.000000000	0.000188067	0.000319708
0.001466997	0.000000000	0.000000000	0.000000000
0.001658731	0.000000000	0.000315457	0.001292732
0.009284303	0.000000000	0.000145206	0.000923492
0.001990143	0.000000000	0.013209181	0.000942863
0.000721508	0.000000000	0.000000000	0.000000000
0.001180744	0.000000000	0.004453821	0.000543833
0.000000000	0.000000000	0.000000000	0.000000000

0.002421609	0.000000000	0.008941272	0.001790831
0.006283994	0.000000000	0.000165125	0.000235479
0.000531150	0.000000000	0.000000000	0.000000000
0.000000000	0.000000000	0.000000000	0.000000000
1.000000000	0.000070600	0.001577304	0.013402389
0.000070600	1.000000000	0.000000000	0.000000000
0.001576524	0.000000000	1.000000000	0.002972788
0.013406299	0.000000000	0.002972788	1.000000000
0.000000000	0.000000000	0.000000000	0.000000000
0.000084900	0.000000000	0.000000000	0.000000000
0.009611910	0.000447099	0.000055500	0.000165411
0.005925300	0.000000000	0.000147551	0.000477213
0.000120868	0.000000000	0.000000000	0.000000000
0.008670520	0.000000000	0.000000000	0.004541326
0.002009302	0.000000000	0.000000000	0.000682687
0.006803102	0.000000000	0.000199433	0.000324767
0.000323415	0.000000000	0.000000000	0.000000000
0.000053300	0.000000000	0.000000000	0.000000000
0.000000000	0.000000000	0.000000000	0.000000000
0.006593301	0.000000000	0.000157122	0.000674056
0.001860482	0.000000000	0.001767113	0.000360946
0.001153391	0.000000000	0.000000000	0.000000000
0.000063400	0.000000000	0.000000000	0.000000000
0.008683083	0.000000000	0.030303030	0.004828889
0.000191388	0.000000000	0.000000000	0.000000000
0.000053400	0.000000000	0.000000000	0.000000000
0.000000000	0.000000000	0.000000000	0.000000000
0.002153588	0.000000000	0.001335368	0.001192098
0.019356637	0.000000000	0.001103220	0.029843644
0.003015065	0.000000000	0.000730536	0.002550770
0.000579239	0.000000000	0.000000000	0.000397509
0.000032100	0.000000000	0.000000000	0.000000000
0.000000000	0.000000000	0.000053600	0.000353438
0.005351461	0.000000000	0.000471809	0.002101313
0.000788188	0.000000000	0.000000000	0.000463714
0.001124813	0.000000000	0.000915081	0.000328030
0.006246987	0.000000000	0.000305390	0.033237410
0.001210501	0.000534688	0.002525961	0.003342884
0.000269424	0.000000000	0.000000000	0.000000000
0.003579727	0.000000000	0.000000000	0.000456591
0.050227690	0.000076300	0.000677894	0.004377865
0.007546988	0.000053200	0.000623886	0.000904317
0.000628187	0.000000000	0.002738362	0.000000000
0.031610492	0.000089400	0.000776870	0.023750719
0.034169291	0.000264178	0.001224577	0.022410437
0.000084800	0.000000000	0.000000000	0.000000000
0.000021400	0.000000000	0.000000000	0.000000000

0.000063600	0.000000000	0.000000000	0.000000000
0.000745916	0.000000000	0.006251699	0.002081406
0.009856958	0.000000000	0.000413831	0.001073153
0.000042400	0.000000000	0.000000000	0.000000000
0.002888576	0.000000000	0.000000000	0.002674308
0.003222394	0.000000000	0.000228228	0.000169132
0.001858557	0.000000000	0.000893256	0.026080743
0.004926483	0.000090200	0.000395961	0.000560538
0.034541984	0.000135890	0.015783932	0.002092142
0.002449068	0.000000000	0.000000000	0.000188929
0.000074300	0.000000000	0.000000000	0.000000000

Mucha-Habermann disease	Multifocal Motor Neuropathy	Multiple sclerosis	Myasthenia gravis	Narcolepsy
0.000000000	0.000000000	0.000326300	0.001886369	0.000308578
0.000000000	0.000000000	0.000000000	0.000000000	0.000000000
0.000000000	0.000000000	0.000301627	0.001251835	0.000377038
0.000000000	0.000089700	0.000422455	0.000391451	0.000051900
0.000000000	0.000097600	0.001728303	0.000944708	0.000156501
0.000000000	0.000000000	0.001337088	0.001131843	0.000000000
0.000000000	0.000000000	0.000000000	0.000000000	0.000000000
0.000000000	0.000000000	0.000000000	0.000000000	0.000000000
0.000000000	0.000000000	0.079679292	0.001520559	0.000101564
0.000000000	0.000000000	0.000866927	0.001241341	0.000000000
0.000000000	0.000000000	0.000034000	0.000000000	0.000000000
0.000000000	0.000000000	0.000112544	0.000312614	0.000000000
0.000000000	0.000000000	0.000000000	0.000000000	0.000000000
0.000000000	0.000000000	0.000000000	0.000000000	0.000000000
0.000000000	0.000000000	0.000033200	0.000241488	0.000000000
0.000000000	0.000000000	0.000034000	0.000000000	0.000000000
0.000000000	0.000000000	0.000545093	0.000752225	0.000000000
0.000000000	0.000000000	0.000021900	0.000930016	0.000000000
0.000000000	0.000081200	0.001520913	0.001326291	0.000101024
0.000000000	0.000000000	0.000000000	0.000000000	0.000000000
0.000000000	0.000000000	0.000056500	0.000000000	0.000000000
0.000000000	0.000000000	0.000000000	0.000105792	0.000000000
0.000000000	0.000000000	0.000000000	0.000104297	0.000000000
0.000000000	0.000066500	0.003683911	0.000749697	0.000039900
0.000000000	0.000000000	0.000098500	0.000737361	0.000000000
0.000000000	0.000000000	0.000185345	0.000634863	0.000000000
0.000000000	0.000000000	0.000000000	0.000000000	0.000000000
0.000000000	0.000000000	0.000238631	0.000041900	0.000000000
0.000000000	0.000000000	0.000021900	0.000000000	0.000000000
0.000000000	0.000000000	0.000056300	0.000000000	0.000000000
0.000000000	0.000000000	0.000215682	0.000260067	0.000000000
0.000000000	0.000000000	0.000000000	0.000000000	0.000000000
0.000000000	0.000000000	0.000022500	0.000157431	0.000000000
0.000000000	0.000000000	0.001377136	0.000195644	0.001217353
0.000000000	0.000000000	0.000033700	0.000103423	0.000000000
0.000000000	0.000000000	0.000759438	0.001636531	0.000000000
0.000000000	0.000000000	0.000112387	0.000880875	0.000000000
0.000000000	0.000000000	0.000320971	0.000408466	0.000000000
0.000000000	0.000000000	0.001369098	0.006971237	0.000079200
0.000000000	0.015963719	0.005223850	0.013488830	0.002032145
0.000000000	0.000570342	0.000670096	0.002756991	0.000000000
0.000000000	0.000113340	0.000580327	0.002021165	0.000000000
0.000000000	0.000000000	0.000033000	0.000000000	0.000000000
0.000000000	0.000000000	0.000206906	0.000000000	0.000000000
0.000000000	0.000935329	0.000158230	0.000835954	0.000159236
0.000000000	0.002032520	0.000101925	0.003175799	0.000000000

0.000629723	0.000000000	0.000065800	0.000000000	0.000000000
0.000000000	0.000000000	0.000162683	0.000942112	0.000000000
0.000000000	0.000000000	0.000000000	0.000000000	0.000000000
0.000000000	0.000000000	0.000000000	0.000000000	0.000000000
0.000000000	0.000085000	0.009617685	0.005928285	0.000120932
0.000000000	0.000000000	0.000447108	0.000000000	0.000000000
0.000000000	0.000000000	0.000055500	0.000147558	0.000000000
0.000000000	0.000000000	0.000165415	0.000477236	0.000000000
1.000000000	0.000000000	0.000000000	0.000000000	0.000000000
0.000000000	1.000000000	0.000370966	0.002551020	0.000000000
0.000000000	0.000370966	1.000000000	0.006407595	0.001373290
0.000000000	0.002551020	0.006407595	1.000000000	0.000246660
0.000000000	0.000000000	0.001373290	0.000246660	1.000000000
0.000000000	0.000000000	0.000000000	0.000259578	0.000000000
0.000000000	0.000712124	0.026520837	0.005181795	0.001592058
0.000000000	0.000000000	0.001356253	0.000587957	0.000027000
0.000000000	0.000000000	0.000055200	0.000430395	0.000000000
0.000000000	0.000000000	0.000022600	0.000000000	0.000000000
0.000000000	0.000000000	0.000000000	0.000000000	0.000000000
0.000000000	0.000000000	0.000434343	0.005350895	0.000000000
0.000000000	0.001615193	0.001876417	0.001629050	0.000274176
0.000000000	0.000000000	0.000433633	0.002581270	0.000000000
0.000000000	0.004383829	0.000055900	0.000405865	0.000000000
0.000000000	0.000000000	0.000367574	0.001098341	0.000000000
0.000000000	0.000000000	0.000056300	0.001467736	0.000000000
0.000000000	0.000000000	0.000034000	0.000429853	0.000000000
0.000000000	0.000000000	0.000000000	0.000270008	0.000000000
0.000000000	0.000000000	0.000185703	0.000825309	0.000000000
0.000000000	0.001564829	0.001181587	0.011744803	0.000000000
0.000000000	0.000000000	0.000636477	0.001377621	0.000000000
0.000000000	0.000000000	0.000160915	0.000340556	0.000000000
0.000000000	0.000000000	0.000000000	0.000000000	0.000000000
0.000000000	0.000054600	0.005384132	0.001076189	0.000100055
0.000000000	0.000171777	0.000919712	0.000512173	0.000000000
0.000000000	0.000000000	0.000055600	0.005158474	0.000000000
0.000000000	0.000000000	0.000076300	0.000136079	0.000000000
0.000000000	0.000000000	0.000161551	0.000172958	0.000000000
0.000000000	0.000000000	0.000122392	0.000297826	0.000000000
0.000000000	0.000000000	0.000021900	0.000092800	0.000000000
0.000000000	0.000000000	0.000356485	0.000511640	0.000000000
0.000014400	0.000035900	0.011560100	0.003224341	0.000097100
0.000000000	0.000061300	0.002673909	0.001334821	0.000079600
0.000000000	0.000000000	0.000055300	0.000000000	0.000000000
0.000071800	0.000000000	0.001632773	0.001908025	0.000148717
0.000000000	0.000225695	0.002929054	0.003459717	0.000131585
0.000000000	0.009895227	0.000415231	0.002584514	0.000293772
0.000000000	0.000000000	0.000260393	0.000000000	0.000000000

0.000000000	0.000000000	0.000112133	0.000154329	0.000000000
0.000000000	0.000000000	0.000033300	0.000000000	0.000000000
0.000000000	0.000338622	0.001296921	0.003927730	0.000155340
0.000000000	0.000000000	0.000056100	0.000980139	0.000000000
0.000000000	0.000565291	0.007577688	0.001842926	0.000813197
0.000000000	0.000038800	0.001889514	0.001157843	0.000088400
0.000000000	0.000000000	0.000033900	0.000000000	0.000000000
0.000000000	0.000000000	0.003984095	0.001071190	0.000067500
0.000062500	0.000097600	0.003277834	0.001136015	0.000025500
0.000000000	0.000000000	0.000623519	0.002455146	0.000000000
0.000000000	0.000000000	0.000123622	0.000103939	0.000000000

Neonatal Lupus	Neuromyelitis optica	Neutropenia	Paroxysmal nocturnal hemoglobinuria
0.000000000	0.000000000	0.000055400	0.000000000
0.000000000	0.000000000	0.000058300	0.000000000
0.000000000	0.000000000	0.000109544	0.000000000
0.000000000	0.000000000	0.000743450	0.000056900
0.000000000	0.000244888	0.000409319	0.000090300
0.003562429	0.001352047	0.000595035	0.001516990
0.000000000	0.000000000	0.000000000	0.000000000
0.000000000	0.000000000	0.000000000	0.000000000
0.000000000	0.004743935	0.000328899	0.000000000
0.000271628	0.000351989	0.000561445	0.000000000
0.000000000	0.000000000	0.000000000	0.000000000
0.000000000	0.000000000	0.000029000	0.000000000
0.000000000	0.000000000	0.000000000	0.000000000
0.000000000	0.000000000	0.000000000	0.000000000
0.000000000	0.000000000	0.000000000	0.000000000
0.000000000	0.000000000	0.000000000	0.000000000
0.000000000	0.000291064	0.000149090	0.000000000
0.000000000	0.000000000	0.000266289	0.000000000
0.000000000	0.000523981	0.000466980	0.000000000
0.000000000	0.000000000	0.000072200	0.000000000
0.000000000	0.000000000	0.000000000	0.000000000
0.000000000	0.000379651	0.000072700	0.003780069
0.001318392	0.000000000	0.000028900	0.000000000
0.000000000	0.000061100	0.001360798	0.000000000
0.000000000	0.000371471	0.000223629	0.000000000
0.008200456	0.000000000	0.000681834	0.000000000
0.000000000	0.000000000	0.000000000	0.000000000
0.000000000	0.000000000	0.000061500	0.000063100
0.000000000	0.000000000	0.000000000	0.000000000
0.000000000	0.000000000	0.000029000	0.000621311
0.000000000	0.000000000	0.000288050	0.000000000
0.000000000	0.000000000	0.000000000	0.000000000
0.000000000	0.000000000	0.001075066	0.001629726
0.000000000	0.000000000	0.000086900	0.000000000
0.000000000	0.000000000	0.000000000	0.000000000
0.000399174	0.000048700	0.002234664	0.000337405
0.000000000	0.000000000	0.000057800	0.000000000
0.000000000	0.000300729	0.000755887	0.000000000
0.000000000	0.000206577	0.001919452	0.000000000
0.000000000	0.002503624	0.000241481	0.000233227
0.000379363	0.000000000	0.000137453	0.000000000
0.000226612	0.000196844	0.008045397	0.011467395
0.000000000	0.000000000	0.000140669	0.000380011
0.000000000	0.000000000	0.000041700	0.000000000
0.000800427	0.000000000	0.000146708	0.000000000
0.000000000	0.000600240	0.000000000	0.000000000

0.000000000	0.000253197	0.000771064	0.000000000
0.000000000	0.000000000	0.000089100	0.000000000
0.000000000	0.000000000	0.000000000	0.000000000
0.000000000	0.000000000	0.000000000	0.000000000
0.008675070	0.002010430	0.006806491	0.000323581
0.000000000	0.000000000	0.000000000	0.000000000
0.000000000	0.000000000	0.000199436	0.000000000
0.004541326	0.000682687	0.000324772	0.000000000
0.000000000	0.000000000	0.000000000	0.000000000
0.000000000	0.000712124	0.000000000	0.000000000
0.000000000	0.026520837	0.001356253	0.000055200
0.000259578	0.005181795	0.000587957	0.000430395
0.000000000	0.001592058	0.000027000	0.000000000
1.000000000	0.000000000	0.000216982	0.000000000
0.000000000	1.000000000	0.000246110	0.000686908
0.000216982	0.000246110	1.000000000	0.000565307
0.000000000	0.000686908	0.000565307	1.000000000
0.000000000	0.000000000	0.000000000	0.000000000
0.000000000	0.000000000	0.000000000	0.000000000
0.000000000	0.000319101	0.000480101	0.000000000
0.000000000	0.000694785	0.012145889	0.000000000
0.000000000	0.000000000	0.000495321	0.000564398
0.000000000	0.000493421	0.000000000	0.000000000
0.000254065	0.000252483	0.000252056	0.000000000
0.000000000	0.000000000	0.000029000	0.000000000
0.000000000	0.000000000	0.000000000	0.000000000
0.000000000	0.000000000	0.000000000	0.000000000
0.000000000	0.000000000	0.000237135	0.000000000
0.000595105	0.000745028	0.000543666	0.000000000
0.000587130	0.000159617	0.000184133	0.000000000
0.000000000	0.000000000	0.000068100	0.000000000
0.000000000	0.000000000	0.000000000	0.000000000
0.000072900	0.000118819	0.001112038	0.000035400
0.000000000	0.000127681	0.000252653	0.000000000
0.000000000	0.000000000	0.001098964	0.002112180
0.000000000	0.000000000	0.000389435	0.000330469
0.000730060	0.000000000	0.000561890	0.000000000
0.000000000	0.000460617	0.000085700	0.000000000
0.000000000	0.000000000	0.000028000	0.000000000
0.000293341	0.000000000	0.000271123	0.000000000
0.000186861	0.000272461	0.004104971	0.000049700
0.000061300	0.001230180	0.000979598	0.000000000
0.000000000	0.000000000	0.000156084	0.000000000
0.000560518	0.000337817	0.000760306	0.000066200
0.007269010	0.005362178	0.001727055	0.000103157
0.000000000	0.001391546	0.000000000	0.000000000
0.000000000	0.000403063	0.000000000	0.000000000

0.000000000	0.000000000	0.000043300	0.000000000
0.000000000	0.000000000	0.000042800	0.000000000
0.000290079	0.000405006	0.005012760	0.001479224
0.000000000	0.000347041	0.000000000	0.000000000
0.000000000	0.116821740	0.000168859	0.000000000
0.000000000	0.000108052	0.001404742	0.000056400
0.005136986	0.000000000	0.000101903	0.000000000
0.000050800	0.000369072	0.000833380	0.000000000
0.000203982	0.000591158	0.003478425	0.000253494
0.000225023	0.000000000	0.000143970	0.000000000
0.000000000	0.000356952	0.000000000	0.000000000

Parry Romberg syndrome	Parsonage-Turner syndrome	Pemphigus	Peripheral neuropathy	Pernicious anemia
0.000000000	0.000000000	0.000408136	0.000510986	0.008752950
0.000000000	0.000000000	0.000000000	0.000100301	0.000000000
0.000000000	0.000000000	0.003410115	0.000082000	0.001887810
0.000000000	0.000000000	0.000436852	0.007221816	0.000102409
0.000000000	0.000000000	0.000393043	0.000305188	0.000536810
0.000000000	0.000000000	0.000370696	0.000559929	0.000175060
0.000000000	0.000000000	0.000000000	0.000000000	0.000000000
0.000000000	0.000000000	0.000000000	0.000000000	0.000000000
0.000000000	0.000000000	0.000000000	0.000298927	0.000000000
0.000000000	0.000000000	0.000747513	0.000229121	0.001159196
0.000000000	0.000000000	0.000000000	0.000000000	0.000000000
0.000000000	0.000000000	0.000000000	0.000000000	0.000000000
0.000000000	0.000000000	0.000000000	0.000000000	0.000000000
0.000000000	0.000000000	0.000000000	0.000000000	0.000000000
0.000000000	0.000000000	0.000305204	0.000000000	0.000000000
0.000000000	0.000000000	0.000000000	0.000000000	0.000000000
0.000000000	0.000000000	0.077445294	0.000119360	0.000000000
0.000000000	0.000000000	0.008619441	0.001719880	0.000000000
0.000000000	0.000000000	0.000519316	0.002190252	0.004953457
0.000000000	0.000000000	0.009331393	0.000000000	0.000000000
0.000000000	0.000000000	0.000000000	0.000149798	0.000000000
0.000000000	0.000000000	0.000442400	0.000199074	0.000000000
0.000000000	0.000000000	0.000000000	0.000146980	0.000000000
0.000000000	0.000000000	0.000635151	0.000814065	0.000355226
0.000000000	0.000000000	0.048036231	0.000436777	0.001658031
0.000000000	0.000000000	0.012102980	0.000473220	0.000899281
0.000000000	0.000000000	0.000000000	0.000000000	0.000000000
0.000000000	0.000000000	0.000074800	0.000061400	0.000000000
0.000000000	0.000000000	0.000216388	0.000000000	0.000000000
0.000000000	0.000000000	0.000344709	0.000196146	0.000000000
0.000000000	0.000000000	0.001164295	0.000948688	0.000272109
0.000000000	0.000000000	0.000000000	0.000552847	0.000000000
0.000000000	0.000000000	0.000174551	0.000000000	0.000000000
0.000000000	0.000000000	0.000000000	0.002075602	0.000000000
0.000000000	0.000000000	0.000000000	0.000146149	0.000000000
0.000000000	0.000000000	0.000399078	0.001374939	0.000253096
0.000000000	0.000000000	0.000512339	0.000000000	0.000275292
0.000000000	0.000000000	0.000620797	0.002458841	0.000134318
0.000000000	0.000000000	0.000595474	0.000128107	0.002409264
0.000000000	0.000470898	0.000470743	0.009815126	0.000238109
0.000000000	0.000000000	0.000980392	0.000000000	0.003509744
0.000000000	0.000000000	0.000986626	0.000485748	0.006495748
0.000000000	0.000000000	0.000146520	0.000312026	0.000000000
0.000000000	0.000000000	0.000000000	0.000171218	0.000296326
0.000000000	0.000000000	0.000114019	0.000000000	0.000152777
0.000000000	0.000000000	0.000000000	0.000100883	0.000000000

0.000000000	0.000000000	0.001293103	0.001013037	0.000000000
0.000000000	0.000000000	0.021162228	0.000234325	0.000420926
0.000000000	0.000000000	0.001330279	0.000000000	0.000439802
0.000000000	0.000000000	0.000000000	0.000000000	0.000000000
0.000053300	0.000000000	0.006586826	0.001861620	0.001153958
0.000000000	0.000000000	0.000000000	0.000000000	0.000000000
0.000000000	0.000000000	0.000157122	0.001767113	0.000000000
0.000000000	0.000000000	0.000674056	0.000360946	0.000000000
0.000000000	0.000000000	0.000000000	0.000000000	0.000000000
0.000000000	0.000000000	0.000000000	0.001615193	0.000000000
0.000022600	0.000000000	0.000434343	0.001876417	0.000433633
0.000000000	0.000000000	0.005350895	0.001629050	0.002581270
0.000000000	0.000000000	0.000000000	0.000274176	0.000000000
0.000000000	0.000000000	0.000000000	0.000000000	0.000000000
0.000000000	0.000000000	0.000319101	0.000694785	0.000000000
0.000000000	0.000000000	0.000480101	0.012145889	0.000495321
0.000000000	0.000000000	0.000000000	0.000000000	0.000564398
1.000000000	0.000000000	0.000000000	0.000000000	0.000000000
0.000000000	1.000000000	0.000000000	0.000201197	0.000000000
0.000000000	0.000000000	1.000000000	0.000065500	0.000579912
0.000000000	0.000201197	0.000065500	1.000000000	0.000767902
0.000000000	0.000000000	0.000579912	0.000767902	1.000000000
0.000000000	0.000000000	0.000578130	0.006590024	0.000000000
0.000000000	0.000000000	0.000949721	0.003303303	0.000594486
0.000000000	0.000000000	0.000000000	0.000098500	0.005133324
0.000000000	0.000000000	0.000000000	0.000000000	0.001526019
0.000000000	0.000000000	0.000000000	0.000000000	0.001238582
0.000000000	0.000000000	0.000210956	0.000303978	0.000716846
0.000000000	0.000000000	0.003974189	0.001301801	0.000735758
0.000000000	0.000000000	0.000161629	0.000182682	0.001916933
0.000000000	0.000000000	0.000251715	0.000000000	0.000000000
0.000000000	0.000000000	0.000000000	0.000000000	0.000000000
0.000000000	0.000000000	0.007117051	0.000339038	0.000396852
0.000000000	0.000000000	0.000507216	0.000196702	0.000000000
0.000000000	0.000000000	0.000553228	0.000000000	0.000853554
0.000000000	0.000000000	0.003055556	0.000172013	0.000200200
0.000395257	0.000000000	0.000000000	0.001728537	0.000000000
0.000000000	0.000000000	0.000159426	0.000000000	0.000000000
0.000000000	0.000000000	0.000000000	0.000000000	0.000000000
0.000000000	0.000000000	0.000295658	0.000000000	0.000208041
0.000000000	0.000000000	0.001678303	0.001064261	0.000822738
0.000000000	0.000000000	0.000915128	0.001244991	0.000366588
0.000000000	0.000000000	0.000230769	0.000000000	0.000000000
0.003423192	0.000000000	0.003196383	0.001076699	0.000967203
0.000000000	0.000000000	0.002236814	0.004198568	0.001932741
0.000000000	0.000000000	0.000000000	0.000097100	0.000955110
0.000000000	0.000000000	0.000000000	0.000000000	0.000000000

0.000000000	0.000000000	0.000000000	0.000000000	0.000000000
0.000000000	0.000000000	0.000000000	0.000000000	0.000000000
0.000000000	0.000000000	0.000879278	0.000328789	0.002403021
0.000000000	0.000000000	0.000000000	0.000000000	0.000000000
0.000000000	0.000000000	0.000147135	0.001564036	0.000218842
0.000000000	0.000000000	0.000991757	0.000469063	0.001121823
0.000000000	0.000000000	0.000000000	0.000249613	0.000000000
0.000411110	0.000000000	0.001134867	0.000601540	0.000133508
0.000133522	0.000017800	0.001459521	0.005092653	0.000245059
0.000236239	0.000000000	0.003976881	0.000216247	0.008426771
0.000000000	0.000000000	0.000000000	0.000000000	0.000000000

POEMS syndrome	Polyarteritis nodosa	Polyglandular syndromes type I	Polyglandular syndromes type II
0.000782014	0.000733071	0.034435262	0.013615734
0.000000000	0.000543331	0.000000000	0.000000000
0.000000000	0.000254108	0.002043470	0.000000000
0.002274943	0.002093686	0.000000000	0.000000000
0.000000000	0.002735722	0.000000000	0.000000000
0.000000000	0.002646993	0.000000000	0.000000000
0.000000000	0.000000000	0.000000000	0.000000000
0.000000000	0.000000000	0.000000000	0.000000000
0.000000000	0.000095800	0.000000000	0.000000000
0.000255951	0.001102698	0.005023022	0.000595859
0.000000000	0.000000000	0.000000000	0.000000000
0.000000000	0.000000000	0.000000000	0.000000000
0.000000000	0.000000000	0.000000000	0.000000000
0.000000000	0.000000000	0.000000000	0.000000000
0.000000000	0.000000000	0.000000000	0.000000000
0.000000000	0.000000000	0.000000000	0.000000000
0.000000000	0.000318573	0.000000000	0.000000000
0.037817137	0.000000000	0.000000000	0.000000000
0.000000000	0.000323813	0.001759843	0.000166757
0.000000000	0.000000000	0.000000000	0.000000000
0.000000000	0.003230583	0.000000000	0.000000000
0.001749271	0.000000000	0.000000000	0.000000000
0.000000000	0.000000000	0.000000000	0.000000000
0.000000000	0.000701536	0.000089000	0.000000000
0.000000000	0.000678229	0.000511378	0.000000000
0.000000000	0.002158408	0.000704060	0.000000000
0.000000000	0.000000000	0.000000000	0.000000000
0.000000000	0.000055100	0.000066900	0.000000000
0.000000000	0.000000000	0.000000000	0.000000000
0.000000000	0.000255820	0.000000000	0.000000000
0.000000000	0.006543826	0.000000000	0.000000000
0.000000000	0.001092448	0.000000000	0.000000000
0.000000000	0.000000000	0.000000000	0.000000000
0.000000000	0.000363071	0.000000000	0.000000000
0.000000000	0.000251541	0.000000000	0.000000000
0.000447759	0.007966556	0.000052200	0.000000000
0.000000000	0.002655539	0.000000000	0.000000000
0.000000000	0.042004422	0.000000000	0.000000000
0.000000000	0.000491586	0.003102948	0.000716369
0.002492694	0.000630228	0.000000000	0.000000000
0.000000000	0.000433952	0.009678056	0.003470716
0.000185673	0.001376526	0.000000000	0.000000000
0.000000000	0.013667660	0.000000000	0.000000000
0.000000000	0.000000000	0.000000000	0.000000000
0.000000000	0.009096911	0.000000000	0.000000000
0.000000000	0.000000000	0.000000000	0.000000000

0.000000000	0.014797041	0.000000000	0.000000000
0.000000000	0.001157474	0.000000000	0.000000000
0.000000000	0.000000000	0.000000000	0.000000000
0.000000000	0.000000000	0.000000000	0.000000000
0.000063400	0.008687443	0.000191487	0.000053500
0.000000000	0.000000000	0.000000000	0.000000000
0.000000000	0.030303030	0.000000000	0.000000000
0.000000000	0.004828889	0.000000000	0.000000000
0.000000000	0.000000000	0.000000000	0.000000000
0.004383829	0.000000000	0.000000000	0.000000000
0.000055900	0.000367574	0.000056300	0.000034000
0.000405865	0.001098341	0.001467736	0.000429853
0.000000000	0.000000000	0.000000000	0.000000000
0.000000000	0.000254065	0.000000000	0.000000000
0.000493421	0.000252483	0.000000000	0.000000000
0.000000000	0.000252056	0.000029000	0.000000000
0.000000000	0.000000000	0.000000000	0.000000000
0.000000000	0.000000000	0.000000000	0.000000000
0.000000000	0.000000000	0.000000000	0.000000000
0.000578130	0.000949721	0.000000000	0.000000000
0.006590024	0.003303303	0.000098500	0.000000000
0.000000000	0.000594486	0.005133324	0.001526019
1.000000000	0.000240269	0.000000000	0.000000000
0.000240269	1.000000000	0.000000000	0.000000000
0.000000000	0.000000000	1.000000000	0.250000000
0.000000000	0.000000000	0.250000000	1.000000000
0.000000000	0.000000000	0.101851852	0.117647059
0.000000000	0.006559275	0.000000000	0.000000000
0.000143875	0.016088279	0.000000000	0.000000000
0.000000000	0.000880639	0.000838123	0.000381194
0.000000000	0.000247872	0.000703482	0.000000000
0.000000000	0.000000000	0.000000000	0.000000000
0.000036100	0.000621677	0.000054800	0.000000000
0.000000000	0.000838270	0.000000000	0.000000000
0.000000000	0.000451060	0.003269309	0.000000000
0.000000000	0.002540220	0.000000000	0.000000000
0.000674423	0.001279536	0.000000000	0.000000000
0.000000000	0.003896848	0.000000000	0.000000000
0.000000000	0.001378495	0.000000000	0.000000000
0.000000000	0.003936015	0.000000000	0.000000000
0.000078800	0.003803384	0.000115117	0.000028900
0.000090700	0.003045161	0.000154102	0.000000000
0.000000000	0.002611250	0.000000000	0.000000000
0.000758856	0.010770794	0.000000000	0.000000000
0.000384996	0.005447993	0.000512353	0.000234124
0.000911162	0.000000000	0.000000000	0.000000000
0.000000000	0.000000000	0.000000000	0.000000000

0.000000000	0.000000000	0.000000000	0.000000000
0.000000000	0.005770536	0.000000000	0.000000000
0.000236563	0.002530138	0.000292383	0.000149716
0.000000000	0.000000000	0.000000000	0.000000000
0.000000000	0.000204019	0.000000000	0.000000000
0.000000000	0.000831457	0.000077900	0.000000000
0.000000000	0.000000000	0.000000000	0.000000000
0.000050200	0.002748098	0.000204155	0.000000000
0.000335716	0.060201473	0.000026600	0.000000000
0.000000000	0.000132083	0.008202403	0.002798734
0.000000000	0.000000000	0.000000000	0.000000000

Polyglandular syndromes type III	Polymyalgia rheumatica	Polymyositis	Primary biliary cirrhosis
0.003067485	0.000692617	0.000728155	0.001298476
0.000000000	0.001911524	0.000464001	0.000000000
0.000415800	0.000369959	0.000633312	0.000240925
0.000000000	0.000944576	0.001437036	0.000594825
0.000000000	0.004880600	0.004505484	0.000547685
0.000000000	0.000709421	0.002702587	0.001683136
0.000000000	0.000000000	0.000000000	0.000000000
0.000000000	0.000000000	0.000000000	0.000000000
0.000000000	0.000116353	0.000151200	0.000139334
0.000302160	0.000403348	0.001827295	0.074500942
0.000000000	0.000000000	0.000000000	0.000000000
0.000000000	0.000000000	0.000149454	0.000236490
0.000000000	0.000000000	0.000000000	0.000000000
0.000000000	0.000000000	0.000000000	0.000000000
0.000000000	0.000000000	0.000000000	0.002513826
0.000000000	0.000000000	0.000000000	0.000000000
0.000000000	0.000225708	0.001767956	0.000530303
0.000000000	0.000310607	0.000127324	0.000000000
0.000334910	0.000331053	0.000741005	0.003947159
0.000000000	0.000000000	0.000000000	0.000000000
0.000000000	0.000000000	0.000153574	0.000000000
0.000000000	0.000000000	0.000000000	0.000000000
0.000000000	0.000735655	0.002251407	0.008506050
0.000000000	0.000272868	0.000527287	0.001482994
0.000000000	0.000000000	0.001890240	0.000820719
0.000000000	0.000286533	0.007188894	0.000441657
0.000000000	0.000000000	0.000000000	0.000000000
0.000000000	0.000000000	0.000047800	0.000000000
0.000000000	0.000000000	0.000000000	0.000000000
0.000000000	0.000728155	0.001345794	0.000000000
0.000000000	0.000876534	0.001450284	0.000486579
0.000000000	0.000550661	0.000000000	0.000000000
0.000000000	0.000000000	0.000377644	0.000481058
0.000000000	0.004704518	0.001248540	0.000301084
0.000000000	0.000000000	0.001480823	0.000931099
0.000035200	0.000232620	0.001499337	0.000837365
0.000000000	0.000000000	0.000370453	0.000816898
0.000000000	0.002451809	0.003419615	0.000308718
0.000565553	0.000307638	0.001000031	0.001514928
0.000000000	0.000363293	0.003353512	0.000110436
0.001547645	0.000511117	0.001640112	0.003131180
0.000000000	0.000516814	0.001385404	0.001469971
0.000000000	0.000324412	0.000713082	0.000190313
0.000000000	0.000000000	0.000000000	0.000000000
0.000000000	0.000497364	0.001295135	0.000000000
0.000000000	0.000000000	0.000780457	0.000000000

0.000000000	0.000465116	0.000954927	0.000463306
0.000000000	0.000000000	0.001269536	0.001842237
0.000000000	0.000000000	0.000391441	0.000480631
0.000000000	0.000000000	0.000000000	0.000000000
0.000000000	0.002154793	0.019366688	0.003016561
0.000000000	0.000000000	0.000000000	0.000000000
0.000000000	0.001335368	0.001103220	0.000730536
0.000000000	0.001192098	0.029843644	0.002550770
0.000000000	0.000000000	0.000000000	0.000000000
0.000000000	0.000000000	0.001564829	0.000000000
0.000000000	0.000185703	0.001181587	0.000636477
0.000270008	0.000825309	0.011744803	0.001377621
0.000000000	0.000000000	0.000000000	0.000000000
0.000000000	0.000000000	0.000595105	0.000587130
0.000000000	0.000000000	0.000745028	0.000159617
0.000000000	0.000237135	0.000543666	0.000184133
0.000000000	0.000000000	0.000000000	0.000000000
0.000000000	0.000000000	0.000000000	0.000000000
0.000000000	0.000000000	0.000000000	0.000000000
0.000000000	0.000210956	0.003974189	0.000161629
0.000000000	0.000303978	0.001301801	0.000182682
0.001238582	0.000716846	0.000735758	0.001916933
0.000000000	0.000000000	0.000143875	0.000000000
0.000000000	0.006559275	0.016088279	0.000880639
0.101851852	0.000000000	0.000000000	0.000838123
0.117647059	0.000000000	0.000000000	0.000381194
1.000000000	0.000000000	0.000000000	0.000000000
0.000000000	1.000000000	0.004769076	0.001174451
0.000000000	0.004769076	1.000000000	0.003797781
0.000000000	0.001174451	0.003797781	1.000000000
0.000000000	0.000000000	0.000339424	0.081404223
0.000000000	0.000000000	0.000000000	0.000000000
0.000036900	0.001079989	0.002538643	0.000663763
0.000000000	0.003600424	0.003075083	0.000431313
0.000000000	0.000000000	0.000346188	0.000000000
0.000000000	0.000574795	0.001048412	0.000354045
0.000000000	0.001244555	0.010387488	0.004623996
0.000000000	0.002771180	0.001397136	0.000319081
0.000000000	0.000772559	0.000254097	0.000832794
0.000000000	0.001297818	0.002874242	0.000146071
0.000021700	0.004514785	0.008199239	0.001851877
0.000125530	0.001138466	0.003477083	0.003300833
0.000000000	0.001087745	0.000405817	0.000000000
0.000000000	0.002092859	0.040440987	0.007672198
0.000000000	0.003807734	0.020606814	0.014661066
0.000000000	0.000000000	0.001401490	0.000000000
0.000000000	0.000000000	0.000000000	0.000000000

0.000000000	0.000000000	0.000000000	0.000000000
0.000000000	0.003670079	0.000276606	0.000209798
0.000000000	0.000516373	0.002466091	0.001233851
0.000000000	0.000000000	0.000000000	0.000000000
0.000000000	0.000000000	0.000585480	0.000478652
0.000000000	0.000443943	0.000868892	0.002247268
0.000000000	0.000535762	0.003627383	0.000495663
0.000077700	0.000644530	0.001722147	0.000302749
0.000000000	0.014004888	0.006843836	0.000677275
0.002091535	0.000349437	0.000869481	0.001839985
0.000000000	0.000000000	0.000000000	0.000000000

Primary sclerosing cholangitis	Progesterone dermatitis	Psoriasis	Psoriatic arthritis	Pure red cell aplasia
0.000224972	0.000000000	0.000293610	0.000204262	0.000000000
0.000000000	0.000000000	0.000036800	0.000539132	0.002370792
0.000409165	0.000000000	0.007901412	0.002122869	0.000458015
0.000583942	0.000000000	0.001478145	0.001196943	0.000116056
0.001255213	0.000000000	0.029968719	0.075841410	0.000093000
0.000507421	0.000000000	0.000355042	0.001020834	0.000318903
0.000000000	0.000000000	0.000000000	0.000000000	0.000000000
0.000000000	0.000000000	0.000000000	0.000000000	0.000000000
0.000000000	0.000000000	0.000766543	0.000121679	0.000000000
0.063211009	0.000000000	0.000676813	0.000921022	0.000478583
0.000000000	0.000000000	0.000036900	0.000181455	0.000000000
0.000000000	0.000000000	0.000054700	0.000000000	0.000000000
0.000000000	0.000000000	0.000000000	0.000000000	0.000000000
0.000000000	0.000000000	0.000000000	0.000000000	0.000000000
0.020568071	0.000000000	0.000053300	0.000000000	0.000000000
0.000000000	0.000000000	0.000000000	0.000000000	0.000000000
0.000189952	0.000000000	0.005282344	0.000367534	0.000000000
0.000000000	0.000000000	0.000122510	0.000000000	0.000614880
0.002605252	0.000000000	0.002445019	0.000924028	0.000117037
0.000000000	0.000000000	0.000145449	0.000000000	0.000000000
0.000000000	0.000000000	0.000000000	0.000178221	0.000000000
0.000000000	0.000000000	0.000000000	0.000000000	0.001326847
0.000000000	0.000000000	0.000000000	0.000000000	0.000000000
0.007990681	0.000000000	0.009203398	0.006445134	0.000043400
0.000482218	0.000885740	0.003588182	0.000283789	0.000000000
0.000000000	0.000000000	0.004020398	0.000831140	0.000000000
0.000000000	0.000000000	0.000000000	0.000000000	0.000000000
0.000000000	0.000068100	0.000192081	0.000049900	0.000000000
0.000000000	0.000000000	0.000035000	0.000000000	0.000000000
0.000000000	0.000000000	0.000145929	0.000431555	0.000000000
0.002698495	0.000000000	0.001757799	0.000711974	0.000000000
0.000000000	0.000000000	0.000000000	0.000000000	0.000000000
0.000000000	0.000000000	0.000036600	0.000000000	0.006666667
0.000115902	0.000000000	0.001283775	0.004924820	0.000000000
0.000000000	0.000000000	0.000054600	0.000000000	0.000000000
0.000307210	0.000000000	0.001218753	0.000443551	0.000221745
0.000000000	0.000000000	0.000054600	0.000000000	0.000000000
0.000591847	0.000000000	0.000271800	0.000621343	0.000000000
0.000204717	0.000000000	0.000858264	0.000529573	0.000000000
0.000000000	0.000000000	0.000217189	0.000094200	0.000000000
0.000632111	0.000000000	0.000649595	0.000261540	0.001117497
0.000456130	0.000000000	0.000564617	0.000191781	0.005562061
0.000000000	0.000000000	0.000299001	0.000440238	0.000000000
0.000000000	0.000000000	0.000069200	0.000000000	0.000000000
0.000000000	0.000000000	0.000526905	0.000114129	0.000000000
0.000000000	0.000000000	0.000000000	0.000000000	0.000000000

0.000000000	0.000000000	0.000857993	0.000575002	0.000000000
0.000458625	0.000193069	0.014560656	0.000664830	0.000581589
0.000000000	0.000000000	0.002171150	0.000000000	0.000000000
0.000000000	0.000000000	0.000000000	0.000000000	0.000000000
0.000579575	0.000032100	0.011622323	0.005335057	0.000788619
0.000000000	0.000000000	0.000000000	0.000000000	0.000000000
0.000000000	0.000000000	0.000053600	0.000471809	0.000000000
0.000397509	0.000000000	0.000353444	0.002101313	0.000463714
0.000000000	0.000000000	0.000000000	0.000000000	0.000000000
0.000000000	0.000000000	0.000054600	0.000171777	0.000000000
0.000160915	0.000000000	0.005384132	0.000919712	0.000055600
0.000340556	0.000000000	0.001076189	0.000512173	0.005158730
0.000000000	0.000000000	0.000100055	0.000000000	0.000000000
0.000000000	0.000000000	0.000072900	0.000000000	0.000000000
0.000000000	0.000000000	0.000118819	0.000127681	0.000000000
0.000068100	0.000000000	0.001112038	0.000252653	0.001098980
0.000000000	0.000000000	0.000035400	0.000000000	0.002112180
0.000000000	0.000000000	0.000000000	0.000000000	0.000000000
0.000000000	0.000000000	0.000000000	0.000000000	0.000000000
0.000251715	0.000000000	0.007117051	0.000507216	0.000553228
0.000000000	0.000000000	0.000339038	0.000196702	0.000000000
0.000000000	0.000000000	0.000396852	0.000000000	0.000853554
0.000000000	0.000000000	0.000036100	0.000000000	0.000000000
0.000247872	0.000000000	0.000621677	0.000838270	0.000451111
0.000703482	0.000000000	0.000054800	0.000000000	0.003269309
0.000000000	0.000000000	0.000000000	0.000000000	0.000000000
0.000000000	0.000000000	0.000036900	0.000000000	0.000000000
0.000000000	0.000000000	0.001079989	0.003600424	0.000000000
0.000339424	0.000000000	0.002538643	0.003075083	0.000346188
0.081404223	0.000000000	0.000663763	0.000431313	0.000000000
1.000000000	0.000000000	0.000490594	0.000251842	0.000000000
0.000000000	1.000000000	0.000055300	0.000000000	0.000000000
0.000490594	0.000055300	1.000000000	0.155196642	0.000000000
0.000251842	0.000000000	0.155196642	1.000000000	0.000000000
0.000000000	0.000000000	0.000000000	0.000000000	1.000000000
0.004887699	0.000000000	0.003408874	0.003126954	0.000000000
0.000205867	0.000000000	0.000323371	0.000709357	0.000000000
0.000000000	0.000000000	0.000304774	0.000558570	0.000000000
0.002337023	0.000000000	0.000104893	0.000358551	0.000000000
0.000000000	0.000000000	0.001121797	0.000845487	0.000000000
0.000306926	0.000000000	0.025070306	0.023726534	0.000363992
0.001086750	0.000000000	0.003481024	0.001973499	0.000119026
0.000556328	0.000000000	0.000231156	0.000924286	0.000000000
0.000243999	0.000000000	0.005132928	0.002910300	0.000135345
0.001823902	0.000000000	0.002823702	0.003722578	0.000854792
0.000000000	0.000000000	0.000000000	0.000000000	0.000000000
0.000000000	0.000000000	0.000000000	0.000000000	0.000000000

0.000000000	0.000000000	0.000000000	0.000000000	0.000000000
0.000000000	0.000000000	0.000089400	0.000315731	0.000000000
0.000280944	0.000000000	0.000635616	0.000227857	0.001940492
0.000000000	0.000000000	0.000000000	0.000000000	0.000000000
0.000000000	0.000000000	0.000123142	0.000220816	0.000000000
0.019920246	0.000039300	0.005940157	0.003881025	0.000095200
0.000370028	0.000000000	0.000073400	0.000535427	0.000000000
0.000803582	0.000000000	0.007656002	0.007115769	0.000049600
0.000384727	0.000017800	0.003710755	0.001990667	0.000193403
0.000304901	0.000000000	0.013212773	0.001057753	0.000202347
0.000000000	0.000000000	0.000091100	0.000000000	0.000000000

Pyoderma gangrenosum	Raynaud's phenomenon	Relapsing polychondritis	Retroperitoneal fibrosis
0.000000000	0.000234549	0.000000000	0.000000000
0.000514403	0.000402253	0.000000000	0.000000000
0.002285302	0.000000000	0.000466128	0.000000000
0.000524413	0.000267924	0.000844816	0.000503511
0.002627046	0.000492752	0.001404692	0.000878040
0.001397917	0.004497458	0.001046699	0.000216747
0.000000000	0.000000000	0.000000000	0.000000000
0.000000000	0.000000000	0.000000000	0.000000000
0.000000000	0.000000000	0.000000000	0.000000000
0.001481189	0.001516638	0.000000000	0.000310688
0.000000000	0.000000000	0.002127660	0.000000000
0.000000000	0.000000000	0.000000000	0.000000000
0.000000000	0.000000000	0.000000000	0.000000000
0.000000000	0.000000000	0.000000000	0.000000000
0.000000000	0.000000000	0.000000000	0.032332564
0.000000000	0.000000000	0.000000000	0.000000000
0.001874724	0.000000000	0.000278242	0.000000000
0.000000000	0.000516662	0.000000000	0.000809848
0.000291875	0.000210571	0.000117559	0.000074300
0.000662983	0.000000000	0.000000000	0.000000000
0.000754717	0.000000000	0.005794302	0.000572738
0.000000000	0.001758156	0.000000000	0.000000000
0.000000000	0.031220073	0.000000000	0.000000000
0.005734812	0.000163425	0.000261040	0.000168830
0.001461347	0.000252080	0.000403551	0.000000000
0.001529265	0.002783156	0.000942685	0.000000000
0.000000000	0.000000000	0.000000000	0.000000000
0.000000000	0.000000000	0.000000000	0.000185529
0.000000000	0.000000000	0.000000000	0.000000000
0.000460511	0.005930319	0.000000000	0.000000000
0.023038605	0.000430385	0.002061202	0.000000000
0.000000000	0.000607780	0.000000000	0.000000000
0.000000000	0.000000000	0.000000000	0.000000000
0.000000000	0.001719436	0.000143730	0.000000000
0.000000000	0.001080108	0.000000000	0.000000000
0.000198649	0.000943934	0.000820836	0.000667824
0.000000000	0.000000000	0.000000000	0.000000000
0.003906250	0.000608180	0.005529226	0.001122431
0.000565956	0.000166237	0.000189717	0.000088900
0.000000000	0.000132705	0.000000000	0.000000000
0.000000000	0.000876520	0.000487488	0.001588142
0.000307671	0.001022664	0.000255857	0.000069500
0.000469778	0.000267738	0.000890869	0.000508302
0.000000000	0.000238237	0.000000000	0.000292912
0.000389257	0.000000000	0.000000000	0.000000000
0.000000000	0.000000000	0.000000000	0.000000000

0.008763977	0.001419904	0.001886397	0.000323154
0.003047232	0.000335503	0.000000000	0.000000000
0.000795925	0.000271444	0.000000000	0.000000000
0.000000000	0.000000000	0.000000000	0.000000000
0.001125440	0.006250385	0.001211177	0.000269573
0.000000000	0.000000000	0.000534688	0.000000000
0.000915248	0.000305390	0.002525961	0.000000000
0.000328084	0.033237410	0.003342884	0.000000000
0.000000000	0.000000000	0.000000000	0.000000000
0.000000000	0.000000000	0.000000000	0.000000000
0.000076300	0.000161554	0.000122395	0.000021900
0.000136091	0.000172965	0.000297841	0.000092800
0.000000000	0.000000000	0.000000000	0.000000000
0.000000000	0.000730060	0.000000000	0.000000000
0.000000000	0.000000000	0.000460688	0.000000000
0.000389446	0.000561898	0.000085700	0.000028000
0.000330524	0.000000000	0.000000000	0.000000000
0.000000000	0.000395257	0.000000000	0.000000000
0.000000000	0.000000000	0.000000000	0.000000000
0.003055768	0.000000000	0.000159426	0.000000000
0.000172043	0.001728751	0.000000000	0.000000000
0.000200220	0.000000000	0.000000000	0.000000000
0.000000000	0.000674423	0.000000000	0.000000000
0.002540698	0.001279645	0.003897295	0.001378631
0.000000000	0.000000000	0.000000000	0.000000000
0.000000000	0.000000000	0.000000000	0.000000000
0.000000000	0.000000000	0.000000000	0.000000000
0.000574960	0.001244710	0.002771728	0.000772678
0.001048477	0.010387488	0.001397136	0.000254097
0.000354076	0.004623996	0.000319081	0.000832794
0.004889406	0.000205910	0.000000000	0.002337598
0.000000000	0.000000000	0.000000000	0.000000000
0.003409111	0.000323388	0.000304791	0.000104899
0.003127172	0.000709357	0.000558570	0.000358551
0.000000000	0.000000000	0.000000000	0.000000000
1.000000000	0.000242072	0.002275744	0.000000000
0.000242072	1.000000000	0.000000000	0.000257202
0.002275744	0.000000000	1.000000000	0.000416927
0.000000000	0.000257202	0.000416927	1.000000000
0.000121810	0.000228584	0.000137722	0.000000000
0.001481607	0.001477394	0.000886176	0.000162614
0.001443001	0.000493719	0.001345211	0.000573263
0.004022385	0.000293212	0.015587847	0.000000000
0.000670477	0.056053154	0.001053920	0.000583695
0.000633992	0.012326006	0.002692370	0.001199520
0.000000000	0.000000000	0.000000000	0.000000000
0.000000000	0.000000000	0.000000000	0.000000000

0.000000000	0.000000000	0.000000000	0.000000000
0.003893215	0.000924357	0.002564833	0.001218769
0.000255711	0.000529769	0.000092700	0.000217590
0.000000000	0.000000000	0.000000000	0.000000000
0.000316006	0.000269869	0.000000000	0.000000000
0.009405554	0.000144462	0.000515464	0.000390356
0.000000000	0.012872084	0.000000000	0.000000000
0.002784388	0.000324037	0.002266557	0.000216128
0.003525775	0.002526945	0.002637568	0.001732348
0.001287001	0.000549235	0.000307062	0.000178859
0.000000000	0.000000000	0.001617469	0.000000000

Rheumatic fever	Rheumatoid arthritis	Sarcoidosis	Scleritis	Scleroderma	Sjogren's syndrome
0.000299832	0.000563174	0.000533303	0.000000000	0.000474924	0.001349333
0.000152393	0.000339277	0.000218177	0.000816327	0.000071300	0.000348473
0.000000000	0.000699692	0.000602542	0.000000000	0.001695856	0.001016401
0.000483889	0.006874006	0.005209227	0.000143649	0.002439551	0.004538088
0.006250774	0.032497582	0.003900004	0.001604916	0.004872365	0.006100074
0.000934500	0.002111606	0.000870773	0.000543394	0.004437368	0.006806435
0.000000000	0.000000000	0.000000000	0.000000000	0.000000000	0.000000000
0.000000000	0.000000000	0.000000000	0.000000000	0.000000000	0.000000000
0.000075100	0.001606775	0.000087700	0.000000000	0.000192502	0.000227724
0.000000000	0.001223609	0.000861394	0.000229832	0.001255364	0.005697640
0.000000000	0.000014400	0.000000000	0.000000000	0.000000000	0.000116891
0.000221060	0.000050400	0.000122948	0.000000000	0.000000000	0.000113193
0.000000000	0.000000000	0.000000000	0.000000000	0.000000000	0.000000000
0.000000000	0.000000000	0.000000000	0.000000000	0.000000000	0.000000000
0.000000000	0.000177923	0.000381971	0.000453823	0.000200033	0.003137583
0.000000000	0.000000000	0.000000000	0.000000000	0.000000000	0.000000000
0.000000000	0.000640312	0.000375637	0.000000000	0.001295376	0.000937542
0.000000000	0.000403417	0.000890012	0.000000000	0.000129820	0.001051209
0.000218311	0.002151389	0.001440507	0.000000000	0.001359223	0.002484564
0.000000000	0.000129313	0.000152802	0.002271252	0.000104610	0.000784358
0.000000000	0.000158681	0.000404179	0.009141494	0.000000000	0.000520111
0.000000000	0.000050500	0.000000000	0.000000000	0.000141874	0.000230203
0.000000000	0.000366540	0.000245987	0.000000000	0.021999713	0.003296578
0.000192638	0.008462499	0.005219207	0.000797397	0.000805880	0.001390548
0.000186498	0.000325098	0.000485215	0.000000000	0.001194782	0.000793179
0.000608643	0.001086504	0.001669260	0.000000000	0.006160955	0.003566891
0.000000000	0.000000000	0.000000000	0.000000000	0.000000000	0.000000000
0.000000000	0.000358297	0.000114711	0.000000000	0.000052700	0.000238333
0.000000000	0.000035400	0.000000000	0.000000000	0.000195122	0.000000000
0.000000000	0.000201430	0.000215133	0.000000000	0.009386512	0.000565931
0.003450060	0.000924551	0.019628157	0.002960332	0.000927730	0.001021925
0.000000000	0.000086600	0.000000000	0.000000000	0.000000000	0.000640279
0.000000000	0.000057600	0.000061700	0.000000000	0.000105835	0.000000000
0.000719137	0.007160495	0.000386408	0.000417624	0.001478511	0.003857413
0.000000000	0.000380983	0.003408358	0.000000000	0.003048033	0.001180306
0.007375698	0.004748001	0.002261932	0.000525869	0.003000510	0.004384950
0.000000000	0.000194083	0.000275693	0.000000000	0.000314718	0.000168672
0.000657246	0.002274047	0.007503940	0.015073459	0.003239741	0.003514314
0.000310472	0.001772633	0.001154057	0.000464382	0.001019390	0.002373592
0.000301477	0.000417180	0.001139006	0.000000000	0.000157332	0.001172462
0.000289302	0.001115148	0.000828226	0.000000000	0.001523869	0.005737127
0.000545795	0.001839302	0.001184813	0.000000000	0.002308125	0.002447520
0.001153329	0.000276519	0.000202417	0.000000000	0.000458610	0.000608766
0.000000000	0.000169039	0.000000000	0.000000000	0.000190718	0.001357839
0.004322767	0.000407149	0.000389965	0.000226270	0.000552117	0.000635593
0.000000000	0.000000000	0.000000000	0.000000000	0.000000000	0.000116891

0.000251462	0.000863729	0.000659896	0.000955840	0.000551715	0.002253944
0.000000000	0.000552423	0.001811681	0.000161512	0.004443151	0.003844021
0.000000000	0.000028400	0.000377085	0.000000000	0.007213050	0.000101942
0.000000000	0.000000000	0.000000000	0.000000000	0.000000000	0.000000000
0.003581689	0.050232575	0.007550937	0.000628536	0.031617860	0.034177536
0.000000000	0.000076300	0.000053200	0.000000000	0.000089500	0.000264178
0.000000000	0.000677942	0.000623904	0.002738362	0.000776896	0.001224577
0.000456651	0.004378178	0.000904344	0.000000000	0.023751523	0.022410437
0.000000000	0.000014400	0.000000000	0.000000000	0.000071800	0.000000000
0.000000000	0.000035900	0.000061300	0.000000000	0.000000000	0.000225695
0.000356492	0.011560616	0.002673976	0.000055300	0.001632801	0.002929110
0.000511656	0.003224527	0.001334874	0.000000000	0.001908066	0.003459815
0.000000000	0.000097100	0.000079600	0.000000000	0.000148721	0.000131590
0.000293341	0.000186872	0.000061300	0.000000000	0.000560518	0.007269010
0.000000000	0.000272478	0.001230248	0.000000000	0.000337827	0.005362426
0.000271126	0.004105150	0.000979618	0.000156086	0.000760314	0.001727075
0.000000000	0.000049700	0.000000000	0.000000000	0.000066200	0.000103157
0.000000000	0.000000000	0.000000000	0.000000000	0.003423192	0.000000000
0.000000000	0.000000000	0.000000000	0.000000000	0.000000000	0.000000000
0.000295658	0.001678393	0.000915149	0.000230769	0.003196383	0.002236814
0.000000000	0.001064335	0.001245088	0.000000000	0.001076767	0.004198913
0.000208041	0.000822783	0.000366598	0.000000000	0.000967203	0.001932741
0.000000000	0.000078800	0.000090700	0.000000000	0.000758856	0.000384996
0.003936213	0.003803620	0.003045318	0.002611534	0.010771107	0.005448221
0.000000000	0.000115124	0.000154107	0.000000000	0.000000000	0.000512353
0.000000000	0.000028900	0.000000000	0.000000000	0.000000000	0.000234124
0.000000000	0.000021700	0.000125534	0.000000000	0.000000000	0.000000000
0.001297899	0.004507953	0.001138531	0.001087942	0.002092926	0.003807922
0.002874242	0.008199677	0.003477161	0.000405817	0.040440987	0.020606814
0.000146071	0.001851979	0.003300917	0.000000000	0.007672198	0.014661066
0.000000000	0.000306947	0.001086838	0.000556483	0.000244014	0.001824069
0.000000000	0.000000000	0.000000000	0.000000000	0.000000000	0.000000000
0.001121848	0.025071774	0.003481186	0.000231169	0.005133117	0.002823822
0.000845487	0.023727837	0.001973545	0.000924286	0.002910300	0.003722578
0.000000000	0.000363992	0.000119026	0.000000000	0.000135345	0.000854792
0.000121810	0.001481607	0.001443001	0.004022385	0.000670477	0.000633992
0.000228584	0.001477394	0.000493719	0.000293212	0.056053154	0.012326006
0.000137722	0.000886176	0.001345211	0.015587847	0.001053920	0.002692370
0.000000000	0.000162614	0.000573263	0.000000000	0.000583695	0.001199520
1.000000000	0.008397364	0.001413507	0.000267094	0.002445531	0.001244953
0.008397364	1.000000000	0.004314492	0.001461134	0.014666447	0.018649838
0.001413507	0.004314492	1.000000000	0.001740721	0.005690933	0.007901051
0.000267094	0.001461134	0.001740721	1.000000000	0.000334549	0.001785433
0.002445531	0.014666447	0.005690933	0.000334549	1.000000000	0.024983367
0.001244953	0.018649838	0.007901051	0.001785433	0.024983367	1.000000000
0.000000000	0.000021500	0.000000000	0.000000000	0.000000000	0.000000000
0.000000000	0.000000000	0.000000000	0.000000000	0.000000000	0.000000000

0.000000000	0.000071800	0.001099237	0.001920000	0.000069600	0.000111694
0.000614502	0.000328220	0.000535539	0.001512097	0.000439754	0.000640376
0.000734259	0.001842239	0.001201038	0.000000000	0.002631302	0.002560471
0.000000000	0.000035900	0.000580525	0.001619171	0.000000000	0.000223889
0.000000000	0.000106310	0.000840190	0.000000000	0.000229629	0.003418367
0.000425083	0.005765927	0.001845422	0.000511015	0.000867391	0.001673405
0.000000000	0.000641923	0.000248756	0.000796495	0.004071429	0.003948438
0.001110678	0.006388123	0.024479394	0.015597315	0.002358598	0.003781847
0.002240622	0.015821295	0.008678570	0.003420442	0.008888921	0.008315667
0.000143493	0.001155303	0.001029039	0.000391198	0.003697686	0.001923231
0.000000000	0.000064700	0.002214295	0.005829904	0.000000000	0.000000000

Stiff person syndrome	Susac's syndrome	Sympathetic ophthalmia	Takayasu's arteritis
0.000417537	0.000000000	0.000000000	0.000000000
0.000000000	0.000000000	0.000000000	0.000943396
0.000000000	0.000000000	0.000000000	0.000000000
0.000000000	0.000000000	0.000000000	0.000522284
0.000000000	0.000000000	0.000387091	0.000697739
0.000171350	0.000183824	0.000000000	0.000956404
0.000000000	0.000000000	0.000000000	0.000000000
0.000000000	0.000000000	0.000000000	0.000000000
0.000203307	0.000000000	0.000000000	0.000000000
0.000000000	0.000000000	0.000000000	0.000000000
0.000000000	0.000000000	0.000000000	0.000000000
0.000000000	0.000000000	0.000000000	0.000000000
0.000000000	0.000000000	0.000000000	0.000000000
0.000000000	0.000000000	0.000000000	0.000000000
0.000000000	0.000000000	0.000000000	0.000000000
0.000000000	0.000000000	0.000000000	0.000000000
0.000000000	0.000000000	0.000000000	0.000000000
0.000000000	0.000000000	0.000000000	0.000000000
0.000201955	0.000000000	0.000000000	0.000155982
0.000000000	0.000000000	0.000000000	0.000000000
0.000000000	0.003773585	0.000000000	0.001814059
0.000000000	0.000000000	0.000000000	0.000000000
0.000000000	0.000000000	0.000000000	0.000000000
0.000000000	0.000000000	0.000000000	0.000955193
0.000714796	0.000000000	0.000000000	0.000000000
0.000000000	0.000000000	0.000000000	0.000000000
0.000000000	0.000000000	0.000000000	0.000000000
0.000000000	0.000000000	0.000000000	0.000000000
0.000000000	0.000000000	0.000000000	0.000000000
0.000000000	0.000000000	0.000000000	0.000000000
0.000000000	0.000000000	0.000000000	0.002488336
0.000000000	0.000000000	0.000000000	0.000000000
0.000000000	0.000000000	0.000000000	0.000000000
0.000000000	0.000000000	0.000000000	0.000142440
0.000000000	0.000000000	0.000000000	0.000000000
0.000000000	0.000000000	0.000000000	0.000494695
0.000000000	0.000000000	0.000000000	0.001118568
0.000000000	0.000000000	0.000000000	0.004188176
0.000295217	0.000000000	0.000147254	0.000094300
0.002387268	0.000000000	0.000000000	0.000000000
0.000370576	0.000000000	0.000000000	0.000000000
0.000000000	0.000000000	0.000075000	0.000000000
0.000000000	0.000000000	0.000000000	0.003693244
0.000000000	0.000000000	0.000000000	0.000000000
0.000393494	0.000000000	0.000000000	0.002591589
0.003577818	0.000000000	0.000000000	0.000000000

0.000000000	0.000000000	0.000000000	0.000000000
0.000000000	0.000000000	0.000000000	0.000000000
0.000000000	0.000000000	0.000000000	0.000000000
0.000000000	0.000000000	0.000000000	0.000000000
0.000084800	0.000021400	0.000063600	0.000746433
0.000000000	0.000000000	0.000000000	0.000000000
0.000000000	0.000000000	0.000000000	0.006251699
0.000000000	0.000000000	0.000000000	0.002081888
0.000000000	0.000000000	0.000000000	0.000000000
0.009895227	0.000000000	0.000000000	0.000000000
0.000415320	0.000260449	0.000112157	0.000033300
0.002585048	0.000000000	0.000154361	0.000000000
0.000293815	0.000000000	0.000000000	0.000000000
0.000000000	0.000000000	0.000000000	0.000000000
0.001391788	0.000403145	0.000000000	0.000000000
0.000000000	0.000000000	0.000043300	0.000042800
0.000000000	0.000000000	0.000000000	0.000000000
0.000000000	0.000000000	0.000000000	0.000000000
0.000000000	0.000000000	0.000000000	0.000000000
0.000000000	0.000000000	0.000000000	0.000000000
0.000097100	0.000000000	0.000000000	0.000000000
0.000955110	0.000000000	0.000000000	0.000000000
0.000911162	0.000000000	0.000000000	0.000000000
0.000000000	0.000000000	0.000000000	0.005771189
0.000000000	0.000000000	0.000000000	0.000000000
0.000000000	0.000000000	0.000000000	0.000000000
0.000000000	0.000000000	0.000000000	0.000000000
0.000000000	0.000000000	0.000000000	0.003670788
0.001401490	0.000000000	0.000000000	0.000276606
0.000000000	0.000000000	0.000000000	0.000209798
0.000000000	0.000000000	0.000000000	0.000000000
0.000000000	0.000000000	0.000000000	0.000000000
0.000000000	0.000000000	0.000000000	0.000089400
0.000000000	0.000000000	0.000000000	0.000315831
0.000000000	0.000000000	0.000000000	0.000000000
0.000000000	0.000000000	0.000000000	0.003893215
0.000000000	0.000000000	0.000000000	0.000924357
0.000000000	0.000000000	0.000000000	0.002564833
0.000000000	0.000000000	0.000000000	0.001218769
0.000000000	0.000000000	0.000000000	0.000614544
0.000021500	0.000000000	0.000071800	0.000328255
0.000000000	0.000000000	0.001099304	0.000535571
0.000000000	0.000000000	0.001920000	0.001512097
0.000000000	0.000000000	0.000069600	0.000439754
0.000000000	0.000000000	0.000111694	0.000640376
1.000000000	0.000000000	0.000000000	0.000000000
0.000000000	1.000000000	0.000000000	0.000000000

0.000000000	0.000000000	1.000000000	0.000000000
0.000000000	0.000000000	0.000000000	1.000000000
0.000144155	0.000000000	0.000000000	0.000000000
0.000000000	0.000000000	0.000000000	0.000000000
0.000544514	0.000000000	0.000000000	0.000000000
0.000000000	0.000000000	0.000038700	0.000609466
0.000000000	0.000000000	0.000000000	0.000000000
0.000000000	0.000077500	0.021475088	0.000966998
0.000044300	0.000115972	0.000318832	0.014125804
0.000666075	0.000000000	0.001104972	0.000000000
0.000000000	0.000000000	0.020242915	0.000000000

Thrombocytopenic purpura	Thyroid eye disease	Transverse myelitis	Ulcerative colitis
0.000252887	0.000000000	0.000000000	0.000293589
0.000247868	0.000000000	0.000000000	0.000039200
0.000406339	0.000000000	0.000000000	0.000559860
0.000495134	0.000000000	0.000000000	0.001586786
0.000404531	0.000000000	0.000222975	0.007014028
0.007205132	0.000000000	0.004697286	0.000358458
0.000000000	0.000000000	0.000000000	0.000000000
0.000000000	0.000000000	0.000000000	0.000000000
0.000059400	0.000000000	0.000543774	0.000263713
0.001667614	0.000000000	0.000215332	0.002699055
0.000000000	0.000000000	0.000000000	0.000078700
0.000000000	0.000000000	0.000000000	0.000000000
0.000000000	0.000000000	0.000000000	0.000000000
0.000000000	0.000000000	0.000000000	0.000000000
0.000451977	0.000000000	0.000000000	0.001286636
0.000000000	0.000000000	0.000000000	0.000000000
0.000236537	0.000000000	0.000000000	0.000641483
0.000828573	0.000000000	0.000000000	0.000074400
0.000663995	0.000000000	0.000112948	0.008229561
0.000000000	0.000000000	0.000000000	0.000038800
0.000000000	0.000000000	0.000000000	0.000137142
0.000886088	0.000000000	0.000000000	0.000000000
0.000097100	0.000000000	0.000000000	0.000000000
0.000608168	0.000044200	0.000042500	0.209246052
0.000172876	0.000000000	0.000333444	0.000760880
0.000255460	0.000000000	0.000000000	0.000239570
0.000000000	0.000000000	0.000000000	0.000000000
0.000285103	0.000000000	0.000000000	0.000450749
0.000000000	0.000000000	0.000000000	0.000372738
0.000145412	0.000000000	0.000000000	0.000000000
0.000408263	0.000000000	0.000000000	0.003539470
0.000099300	0.000000000	0.000000000	0.000000000
0.012804667	0.000000000	0.000000000	0.000078000
0.000062300	0.000000000	0.000000000	0.000286319
0.000000000	0.000000000	0.000000000	0.000232833
0.003269726	0.000000000	0.000184700	0.001089629
0.001061725	0.000000000	0.000000000	0.000038800
0.000811431	0.001592864	0.000000000	0.000913737
0.001964536	0.012446480	0.000000000	0.000742815
0.003646127	0.000000000	0.011861665	0.000327638
0.001110471	0.003527664	0.000000000	0.000471878
0.045036364	0.000000000	0.000422595	0.002192551
0.001059416	0.000000000	0.000000000	0.000392957
0.000084700	0.000000000	0.000000000	0.000147116
0.001961006	0.000000000	0.000000000	0.000104626
0.000000000	0.000000000	0.000000000	0.000000000

0.000784450	0.000000000	0.000687521	0.000595681
0.000564859	0.000000000	0.000000000	0.000542157
0.000000000	0.000000000	0.000000000	0.000037500
0.000000000	0.000000000	0.000000000	0.000000000
0.009862496	0.000042400	0.002890565	0.003224840
0.000000000	0.000000000	0.000000000	0.000000000
0.000413831	0.000000000	0.000000000	0.000228315
0.001073201	0.000000000	0.002675330	0.000169198
0.000000000	0.000000000	0.000000000	0.000000000
0.000338622	0.000000000	0.000565451	0.000038800
0.001297149	0.000056100	0.007579367	0.001882804
0.003928141	0.000980342	0.001843362	0.001158228
0.000155346	0.000000000	0.000813386	0.000088400
0.000290093	0.000000000	0.000000000	0.000000000
0.000405022	0.000347102	0.116856256	0.000108091
0.005012874	0.000000000	0.000168867	0.001404991
0.001479224	0.000000000	0.000000000	0.000056400
0.000000000	0.000000000	0.000000000	0.000000000
0.000000000	0.000000000	0.000000000	0.000000000
0.000879336	0.000000000	0.000147167	0.000992096
0.000328864	0.000000000	0.001564735	0.000469250
0.002403021	0.000000000	0.000218866	0.001122197
0.000236563	0.000000000	0.000000000	0.000000000
0.002530233	0.000000000	0.000204061	0.000831745
0.000292383	0.000000000	0.000000000	0.000077900
0.000149716	0.000000000	0.000000000	0.000000000
0.000000000	0.000000000	0.000000000	0.000000000
0.000516396	0.000000000	0.000000000	0.000444107
0.002466091	0.000000000	0.000585518	0.000869153
0.001233851	0.000000000	0.000478698	0.002248001
0.000280967	0.000000000	0.000000000	0.019927901
0.000000000	0.000000000	0.000000000	0.000039100
0.000635728	0.000000000	0.000123172	0.005941982
0.000227887	0.000000000	0.000220897	0.003882482
0.001940492	0.000000000	0.000000000	0.000095200
0.000255711	0.000000000	0.000316006	0.009407478
0.000529769	0.000000000	0.000269869	0.000144490
0.000092700	0.000000000	0.000000000	0.000515572
0.000217590	0.000000000	0.000000000	0.000390436
0.000734282	0.000000000	0.000000000	0.000425163
0.001842413	0.000035900	0.000106321	0.005761374
0.001201085	0.000580560	0.000840239	0.001845713
0.000000000	0.001619171	0.000000000	0.000511122
0.002631302	0.000000000	0.000229629	0.000867513
0.002560471	0.000223889	0.003418367	0.001673677
0.000144155	0.000000000	0.000544514	0.000000000
0.000000000	0.000000000	0.000000000	0.000000000

0.000000000	0.000000000	0.000000000	0.000038700
0.000000000	0.000000000	0.000000000	0.000609466
1.000000000	0.000000000	0.000309570	0.001191354
0.000000000	1.000000000	0.000000000	0.000000000
0.000309570	0.000000000	1.000000000	0.000037400
0.001191354	0.000000000	0.000037400	1.000000000
0.000098800	0.000000000	0.000000000	0.000000000
0.000479477	0.000101304	0.000654403	0.003812416
0.003646273	0.000062000	0.000820151	0.003968229
0.000930832	0.000000000	0.000184894	0.000714894
0.000000000	0.000000000	0.000000000	0.000097100

Undifferentiated connective tissue disease	Uveitis	Vasculitis	Vitiligo
0.000000000	0.000070800	0.000207345	0.008279824
0.000000000	0.000206132	0.000187171	0.000000000
0.000000000	0.000624508	0.000282951	0.033282295
0.000060600	0.002069548	0.004984012	0.000270084
0.000299237	0.022089552	0.003936167	0.000828918
0.001985380	0.001158584	0.006053299	0.000372162
0.000000000	0.000000000	0.000000000	0.000000000
0.000000000	0.000000000	0.000000000	0.000000000
0.000000000	0.001914242	0.000174995	0.000091300
0.000000000	0.000599427	0.000810989	0.002329246
0.000000000	0.000051700	0.000107053	0.000000000
0.000000000	0.000050900	0.000017800	0.000000000
0.000000000	0.000000000	0.000000000	0.000000000
0.000000000	0.000000000	0.000044600	0.000000000
0.000000000	0.000098100	0.000280353	0.000000000
0.000000000	0.000465814	0.000026800	0.000000000
0.000000000	0.000249994	0.000596552	0.003103983
0.000000000	0.000168338	0.000321873	0.000000000
0.000124023	0.000866913	0.000965991	0.002450749
0.000000000	0.000862484	0.000079800	0.000221877
0.000000000	0.001235299	0.001855552	0.000000000
0.000000000	0.000000000	0.000071200	0.000000000
0.002808989	0.000127311	0.000248421	0.000341219
0.000000000	0.004780741	0.003642976	0.000764453
0.000000000	0.000335169	0.000555937	0.002386424
0.001762336	0.000451135	0.001204537	0.003519011
0.000000000	0.000000000	0.000000000	0.000000000
0.000000000	0.000059000	0.000233829	0.000080400
0.000000000	0.000000000	0.000034800	0.000000000
0.000000000	0.000000000	0.000213044	0.000793111
0.000000000	0.011653263	0.006398632	0.001103318
0.000000000	0.000000000	0.000731451	0.000000000
0.000000000	0.000102239	0.000088900	0.000345543
0.000635981	0.000512649	0.000765826	0.000295654
0.002400000	0.000152273	0.000203924	0.000000000
0.000104915	0.001733448	0.030559152	0.000277393
0.000000000	0.000050800	0.001242832	0.000000000
0.000000000	0.003286666	0.071453360	0.000000000
0.000101235	0.001140921	0.001754587	0.003946936
0.000000000	0.000511289	0.001211387	0.000216521
0.000000000	0.000442231	0.000326612	0.006259027
0.000192079	0.000498753	0.002367631	0.000978764
0.000632511	0.000508487	0.021622007	0.000459897
0.000000000	0.000047300	0.000077800	0.000000000
0.000000000	0.000974163	0.048161043	0.000270929
0.000000000	0.000000000	0.000000000	0.000000000

0.000000000	0.001395909	0.027580969	0.000448109
0.000000000	0.001047099	0.001442800	0.010707584
0.000000000	0.000048500	0.000122154	0.006351579
0.000000000	0.000000000	0.000000000	0.000000000
0.001859769	0.004929712	0.034533846	0.002450617
0.000000000	0.000090200	0.000135897	0.000000000
0.000893256	0.000396000	0.015784777	0.000000000
0.026090064	0.000560606	0.002092270	0.000188947
0.000000000	0.000000000	0.000062500	0.000000000
0.000000000	0.000000000	0.000097600	0.000000000
0.000033900	0.003984821	0.003278245	0.000623642
0.000000000	0.001071341	0.001136102	0.002455517
0.000000000	0.000067500	0.000025500	0.000000000
0.005141388	0.000050800	0.000203995	0.000225048
0.000000000	0.000369114	0.000591194	0.000000000
0.000101906	0.000833427	0.003478580	0.000143973
0.000000000	0.000000000	0.000253508	0.000000000
0.000000000	0.000411153	0.000133529	0.000236239
0.000000000	0.000000000	0.000017800	0.000000000
0.000000000	0.001135005	0.001459616	0.003977303
0.000249725	0.000601674	0.005093236	0.000216318
0.000000000	0.000133520	0.000245071	0.008426771
0.000000000	0.000050200	0.000335734	0.000000000
0.000000000	0.002748400	0.060195783	0.000132092
0.000000000	0.000204175	0.000026600	0.008202403
0.000000000	0.000000000	0.000000000	0.002798734
0.000000000	0.000077700	0.000000000	0.002091535
0.000535906	0.000644607	0.014005750	0.000349467
0.003627383	0.001722282	0.006844168	0.000869481
0.000495663	0.000302775	0.000677308	0.001839985
0.000370165	0.000803692	0.000384754	0.000304948
0.000000000	0.000000000	0.000017800	0.000000000
0.000073500	0.007657418	0.003711181	0.013215572
0.000535619	0.007116930	0.001990829	0.001057977
0.000000000	0.000049600	0.000193412	0.000202347
0.000000000	0.002784521	0.003525928	0.001287001
0.012872084	0.000324052	0.002527054	0.000549235
0.000000000	0.002266670	0.002637684	0.000307062
0.000000000	0.000216138	0.001732423	0.000178859
0.000000000	0.001110743	0.002240730	0.000143499
0.000641992	0.006388740	0.015822579	0.001155421
0.000248772	0.024465901	0.008678996	0.001029091
0.000796495	0.015598093	0.003420592	0.000391198
0.004071429	0.002358669	0.008889242	0.003697686
0.003948438	0.003781984	0.008315992	0.001923231
0.000000000	0.000000000	0.000044300	0.000666075
0.000000000	0.000077500	0.000115972	0.000000000

0.000000000	0.021475088	0.000318832	0.001104972
0.000000000	0.000966998	0.014125804	0.000000000
0.000098800	0.000479477	0.003646273	0.000930832
0.000000000	0.000101304	0.000062000	0.000000000
0.000000000	0.000654403	0.000820151	0.000184894
0.000000000	0.003812416	0.003968229	0.000714894
1.000000000	0.000000000	0.000267142	0.000000000
0.000000000	1.000000000	0.075784433	0.003465048
0.000267142	0.075784433	1.000000000	0.000358324
0.000000000	0.003465048	0.000358324	1.000000000
0.000000000	0.017804922	0.000354893	0.005103777

Vogt-Koyanagi-Harada Disease
0.000000000
0.000000000
0.000000000
0.000000000
0.000244236
0.000000000
0.000000000
0.000000000
0.000000000
0.000000000
0.000000000
0.000000000
0.000000000
0.000000000
0.000000000
0.002159827
0.000000000
0.000000000
0.000000000
0.000000000
0.000000000
0.000000000
0.000000000
0.000000000
0.000000000
0.000000000
0.000000000
0.000000000
0.000000000
0.000000000
0.000000000
0.000000000
0.000000000
0.000000000
0.000000000
0.000000000
0.000000000
0.000000000
0.000099100
0.000268288
0.000381316
0.000000000
0.000000000
0.000000000
0.000000000
0.000000000

0.000000000
0.000000000
0.000000000
0.000000000
0.000074400
0.000000000
0.000000000
0.000000000
0.000000000
0.000000000
0.000123648
0.000103961
0.000000000
0.000000000
0.000357015
0.000000000
0.000000000
0.000000000
0.000000000
0.000000000
0.000000000
0.000000000
0.000000000
0.000000000
0.000000000
0.000000000
0.000000000
0.000000000
0.000000000
0.000000000
0.000000000
0.000000000
0.000091100
0.000000000
0.000000000
0.000000000
0.000000000
0.001617469
0.000000000
0.000000000
0.000064700
0.002214431
0.005829904
0.000000000
0.000000000
0.000000000
0.000000000

0.020242915
0.000000000
0.000000000
0.000000000
0.000000000
0.000097100
0.000000000
0.017804922
0.000354893
0.005103777
1.000000000
