# Supplementary material for: Autoimmune Disease Classification Based on PubMed Text Mining
Source: J Clin Med. 2022 Jul 26;11(15):4345. doi: 10.3390/jcm11154345 (PMC9369164; doi:10.3390/jcm11154345)
Supplement: Supplementary file 1 [file jcm-11-04345-s001.zip › jcm-1744611-supplementary Table S2.pdf]

Supplementary Table S2.

Autoimmune Disease	Gender (% Female)	Onset Age (Year)	Prevalence in Population	Affected System
Eosinophilic esophagitis	25	NA	0.0005	Gastrointestinal
Autoimmune pancreatitis	35	55	0.00001	Multiple
Retroperitoneal fibrosis	25	55	0.00001	Multiple
Castleman disease	45	55	0.000002	Multiple
POEMS syndrome	35	55	0.000003	Multiple
Guillain–Barré Syndrome	40	25, 65	0.00001	Neuronal
Miller Fisher syndrome	45	45	0.0000001	Neuronal
Chronic inflammatory demyelinating polyneuropathy	35	55	0.00001	Neuronal
Multifocal Motor Neuropathy	35	45	0.00001	Neuronal
Relapsing polychondritis	50	45	0.000001	Musculoskeletal
Scleritis	65	45	0.00004	Eye
Thyroid eye disease	80	45	0.0001	Eye
Sympathetic ophthalmia	50	45	0.0001	Eye
Vogt–Koyanagi–Harada Disease	65	NA	0.0000001	Eye, Neuronal
Goodpasture's disease	30	NA	0.00001	Kidneys and Lungs
Fibrosing alveolitis	35	55	0.00001	Kidneys and Lungs
Congenital heart block	50	0	0.0001	Cardiovascular
Neonatal Lupus	50	0	0.0001	Cardiovascular
Eosinophilic fasciitis	40	45	<0.0000001	Cutaneous
Stiff person syndrome	35	NA	0.0000001	Neuronal
Undifferentiated connective tissue disease	90	45	0.00001	Cutaneous
Cold agglutinin disease	65	60	0.00001 *	Hematopoietic
Parry Romberg syndrome	60	10	0.000004	Cutaneous
Autoimmune inner ear disease	65	25	0.0000001	Neuronal
Cogan's syndrome	55	35	0.0000001	Multiple
Lambert–Eaton syndrome	33	35	0.000004	Neuronal
Susac syndrome	80	30	0.000001	Cardiovascular
Autoimmune orchitis	0	25	0.0000001	Endrocrine
Autoimmune oophoritis	100	35	0.0000001	Endrocrine
Progesterone dermatitis	95	25	<0.0000001	Cutaneous
Mucha–Habermann disease	35	10	<0.0000001	Cutaneous
Type 1 diabetes mellitus	40	5	0.001	Endrocrine
Graves' disease	80	25	0.01	Endrocrine
Hashimoto's thyroiditis	90	40	0.01	Endrocrine
Pernicious anemia	60	35	0.001	
Polyglandular syndrome 3	85	45	0.0000001	Endrocrine
Polyglandular syndrome 2	80	30	0.00001	Endrocrine
Addison's disease	60	25	0.0001	Endrocrine
Polyglandular syndrome 1	60	5	0.00001	Endrocrine
Dermatitis herpetiformis	35	25	0.0001	Cutaneous
Bullous pemphigoid	50	65	0.00001	Cutaneous
Cicatricial pemphigoid	75	45	0.000001	Cutaneous

Pemphigus foliaceus	50	45	0.000001	Cutaneous
Pemphigus	60	45	0.00001	Cutaneous
Discoid lupus erythematosus	65	25	0.0001	Cutaneous
Lichen planus	60	25	0.01	Cutaneous
Alopecia areata	50	25	0.001	Cutaneous
Vitiligo	50	15	0.001	Cutaneous
Celiac disease	65	5	0.01	Gastrointestinal
Crohn's disease	45	25	0.0001	Gastrointestinal
Ulcerative colitis	60	25	0.0001	Gastrointestinal
Autoimmune hepatitis type 1	80	25	0.0001	Gastrointestinal
Autoimmune hepatitis type 2	85	25	0.00001	Gastrointestinal
Primary biliary cholangitis	90	45	0.0001	Gastrointestinal
Primary sclerosing cholangitis	35	25	0.0001	Gastrointestinal
Inclusion body myositis	50	25	0.000001	Musculoskeletal
Dermatomyositis	65	35	0.0001	Musculoskeletal
Polymyositis	65	35	0.0001	Musculoskeletal
CREST syndrome	80	35	0.0001	Multiple
Mixed connective tissue disease	80	15	0.00001	Multiple
Scleroderma	90	40	0.0001	Multiple
Psoriasis	50	35	0.02	Cutaneous
Sarcoidosis	65	25	0.0001	Multiple
Sjögren's syndrome	90	55	0.0001	Multiple
Rheumatoid arthritis	75	40, 65	0.01	Multiple
Systemic lupus erythematosus	80	30	0.0001	Multiple
Amyloidosis	NR	65	0.000001	Multiple
Glomerulonephritis	35	10	0.00001	Kidneys
Kawasaki disease	40	5	0.0001	Cardiovascular
Vasculitis	65	25	0.00001	Cardiovascular
Uveitis (Behçet's disease)	35	40	0.0001	Eye
Polyarteritis nodosa	35	25	0.0001	Cardiovascular
Granulomatosis with polyangiitis	45	40	0.00001	Cardiovascular
Microscopic polyangiitis	50	45	0.00001	Cardiovascular
Temporal arteritis	85	65	0.0001	Cardiovascular
Takayasu arteritis	80	30	0.000001	Cardiovascular
Still's disease	60	20, 40	0.00001	Multiple
Narcolepsy	50	15, 35	0.0001	Neuronal
Myasthenia gravis	70	30, 65	0.0001	Neuronal
Multiple sclerosis	60	30, 50+	0.001	Neuronal
Alzheimer's disease (autoimmune caused)	60	65	0.001	Neuronal
Hashimoto's encephalopathy	90	45	0.00001	Neuronal
Optic neuritis	65	35	0.00001	Neuronal
Neuromyelitis optica	75	45	0.00001	Neuronal
Transverse myelitis	60	15, 35	0.00001	Neuronal
Neutropenia	55	5, 25	0.00001	Hematopoietic
Antiphospholipid syndrome	75	45	0.0001	Hematopoietic
Paroxysmal nocturnal hemoglobinuria	60	35	0.000001	Hematopoietic

Evans syndrome	60	55	0.0000001	Hematopoietic
Hemolytic anemia	60	65	0.00001	Hematopoietic
Thrombocytopenic purpura	70	55	0.001	Hematopoietic
Felty’s syndrome	75	60	0.00001	Hematopoietic

* 0.00001 (cold weather)–0.000001 (warm weather); NR—Not reported; NA—Not applicable.
